# MEMS-Based Tactile Sensors: Materials, Processes and Applications in Robotics

**DOI:** 10.3390/mi13122051

**Published:** 2022-11-23

**Authors:** Ilker S. Bayer

**Affiliations:** Smart Materials, Istituto Italiano di Tecnologia, Via Morego 30, 16163 Genova, Italy; ilker.bayer@iit.it; Tel.: +90-380-387-6699

**Keywords:** MEMS, sensors, robotics, triboelectric, capacitance, tactile sensing, hardness

## Abstract

Commonly encountered problems in the manipulation of objects with robotic hands are the contact force control and the setting of approaching motion. Microelectromechanical systems (MEMS) sensors on robots offer several solutions to these problems along with new capabilities. In this review, we analyze tactile, force and/or pressure sensors produced by MEMS technologies including off-the-shelf products such as MEMS barometric sensors. Alone or in conjunction with other sensors, MEMS platforms are considered very promising for robots to detect the contact forces, slippage and the distance to the objects for effective dexterous manipulation. We briefly reviewed several sensing mechanisms and principles, such as capacitive, resistive, piezoresistive and triboelectric, combined with new flexible materials technologies including polymers processing and MEMS-embedded textiles for flexible and snake robots. We demonstrated that without taking up extra space and at the same time remaining lightweight, several MEMS sensors can be integrated into robotic hands to simulate human fingers, gripping, hardness and stiffness sensations. MEMS have high potential of enabling new generation microactuators, microsensors, micro miniature motion-systems (e.g., microrobots) that will be indispensable for health, security, safety and environmental protection.

## 1. Introduction

### 1.1. Principles of Tactile Sensing: A Summary

The human “sense of touch,” can be briefly and somewhat roughly defined as tactile sensing [1]. Tactile sensing and grasping an object are closely related and physiologically connected in humans [2]. For instance, an experiment conducted on a number of volunteers with anesthetized skin of the fingertips showed significant difficulty in maintaining a stable grasp of objects [3]. “Sense of touch” permits evaluating object properties such as the size, shape, texture and temperature, which in turn are utilized to detect slip and to guide the fingers to contain objects and to develop awareness on what to do with the object in question [4]. To this day, the mechanisms that drive mechanical hypersensitivity and mechanical sensing have not been unlocked completely [5]. For instance, the star-nosed mole (Condylura cristata), a small mole found in moist, low areas in the northern parts of North America, possesses a centimeter-sized touch organ (the star of tentacles on its face) that is decorated with 100,000 nerve fibers, called mechanonociceptors. This number of nerve fibers is five times the number of fibers on a human hand [6]. Latest studies have shown that certain ion channels along with other signaling molecules are involved in touch sensation [7]. Thus far, however, these studies provide only a small window into the complex machinery of mechanosensation [8].

Can humans distinguish between two surfaces that differ by a single layer of molecules at the surface merely with the sense of touch? The answer is yes, according to the work of Carpenter et al. [9] who showed that indeed humans can discriminate such surfaces and can “read” patterns of hydrophobicity in the form of characters in the ASCII alphabet [9]. Humans achieve this by monitoring the forces produced while sliding a finger along surfaces that interact with the mechanoreceptors of the skin to allow the brain to discriminate surfaces that differ only by surface chemistry [10]. Actually, the human “sense of touch” is a very complicated biochemical process. On the cell surface, bundles of fatty lipids exist that act as compartments to keep certain enzymes from mixing with their binding partners. Disrupting the morphology of these bundles through touch, also known as mechanosensation, the enzymes will mix with their partners and react, triggering a signal that communicates the touch to responsive proteins in the cell [11,12].

**Figure 1:**
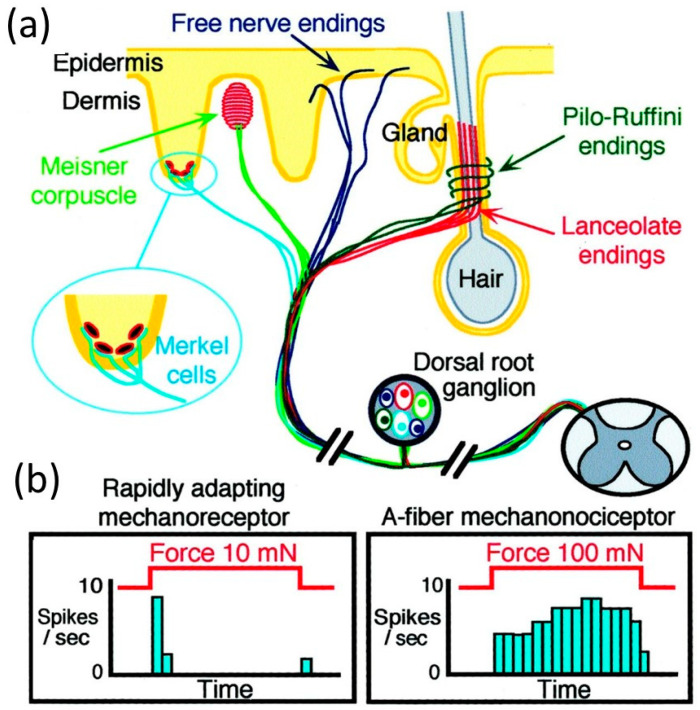
(**a**) Some specialized cutaneous mechanosensory structures. (**b**) Examples of response by two mechanoreceptor fiber types. Reprinted/adapted with permission from Ref. [12]. Copyright 2002, Elsevier.

At the anatomic level, there are several specialized cutaneous sensory structures, such as Meissner corpuscles, Merkel cell-neurite complexes, lanceolate and pilo-Ruffini fibers surrounding hair follicles, and free nerve endings as shown in Figure 1a. Rapidly adapting mechanoreceptors possess large, myelinated fibers, and respond to very light (low threshold) touch. When an excessive constant stimulus is applied, they respond rapidly during movement of the skin, but show no sustained activity in the continued presence of the stimulus (Figure 1b). Rapidly adapting mechanoreceptors include nerve endings in Meissner corpuscles and lanceolate fibers. In contrast, mechano-nociceptor (peripheral endings of primary sensory neurons that are activated only when harmful mechanical stimuli are applied) fibers respond to high threshold mechanical stimuli, such as a pinch, and adapt only slowly to a constant stimulus (Figure 1b). AM fibers include some free nerve endings [12].

To simulate sensing and eventually grasping by a robotic hand, for instance, the most useful parameters to identify are the surface normal, the angle and magnitude of a force at a contact, and whether the finger is touching a corner or edge. These parameters are a subset of those required to recognize general features. This means using solid mechanics and contact theory efficiently to design a tactile sensor [13,14].

**Figure 2:**
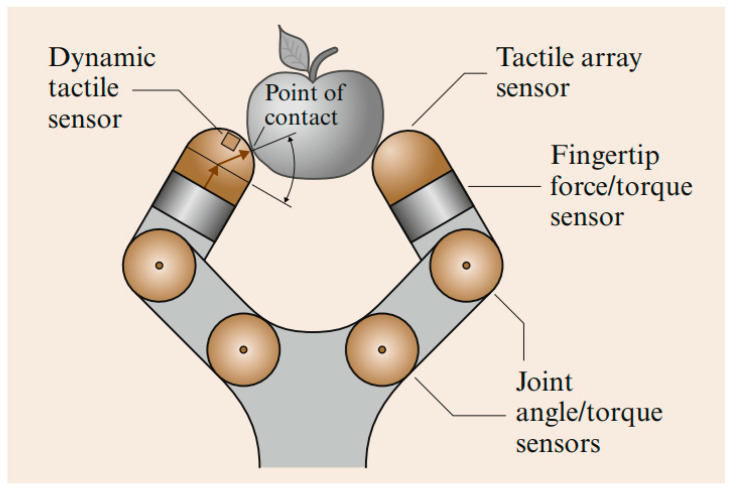
Robot hand with fingertip force and tactile sensing. Information from the force sensors can be combined with knowledge of fingertip geometry to estimate contact location, referred to as intrinsic tactile sensing. Reprinted/adapted with permission from Ref. [14]. Copyright 2008, Springer.

Many robots employ a basic proximity or touch device that detects contact events (see Figure 2). However, such basic “tactile bump sensors” are binary switches that play down the nature of tactile sensing and do not reflect the complexity of skin-based mechanisms nor the sophistication of the many tactile arrays and other devices developed for other applications [15]. The classical robot tactile sensing has been focused on the static perception of object shape with tactile array sensors [16]. In contrast, dynamic tactile sensing is more human-like and is defined as sensing during motion for perception of high spatial and temporal frequencies [17]. Typical applications are sensing fine surface features and monitoring contact conditions for dexterous manipulation [18]. The stress rate sensor is a famous example of a dynamic tactile sensor that uses piezoelectric polymer transducers to measure the changes in stress induced in the sensor’s rubber skin as it traverses small surface features (≤10 μm) and textures. The signals are inferred with the aid of a solid mechanics model of the contact interaction and a linear deconvolution filter [19]. Largely, dynamic tactile sensing comprises several categories of sensors that are either intended to detect motion or incipient motion (slippage), or that exploit the motion of the fingertips to produce results [20]. Other types of dynamic tactile sensors operate in actively stimulated mode, and monitor a change in impedance as they contact objects or surfaces. Finally, there are tactile array sensors that, while not intrinsically designed to detect or use motion, have an adequately fast mechanical response, and can be sampled rapidly enough, to provide dynamic information as contact conditions change [21].

**Figure 3:**
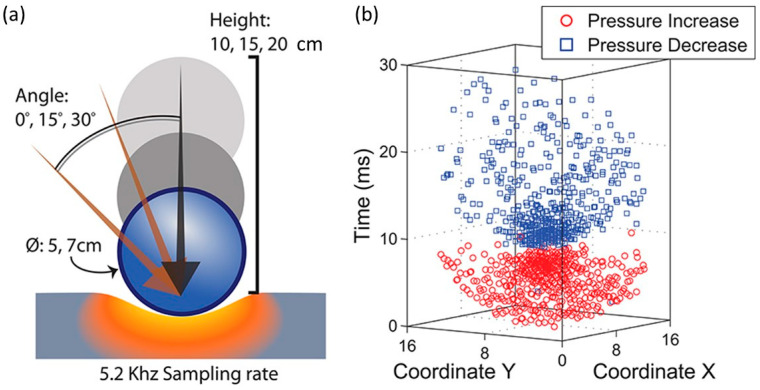
(**a**) Ball dropped on sensor substrate under various physical conditions. (**b**) Spatiotemporal events captured by sensor array. Reprinted/adapted with permission from Ref. [22]. Copyright 2017, Frontiers.

For instance, Lee et al. [22] implemented a novel 4096-element tactile sensor array with a 5.2 kHz sampling frequency in which they demonstrated the classification of transient impact events while utilizing 20 times less communication bandwidth compared to frame-based representations as shown in Figure 3. Figure 3a depicts a graphical sketch of the various experimental scenarios. For each parameter combination, the authors performed 100 trials. Figure 3b shows collected data from a representative impact stimulus from a 5 cm sphere dropped from a height of 10 cm with a 0° slant angle. Data corresponding to increased sampling periods were generated by quantizing the timestamps of the events into bins of lower temporal precision. Soft tactile sensor systems for the localization of sliding movements on a large contact surface using accelerometers are also being developed. These sensors integrate a polymeric soft construct with three-axis accelerometers. Based on the output responses of the accelerometer, the sensor system localizes the sliding motion. Such sensors can detect the sliding movements, including the sliding directions, velocity and localization of an object [23].

### 1.2. Types of Tactile Sensors: A Brief Look

Even though a large majority of tactile sensors are being developed using micro-electro-mechanical-systems (MEMS) technology today in which main components include either polymer-based with organic substrates or silicon-based sensors, before presenting the detailed MEMS tactile sensing technology, it is important to briefly review the more common and standard tactile sensors and their principles [24]. Tactile sensors are defined as devices that can measure a property of an object or contact event through physical contact or between the sensor and the object. A summary of typical and classical technologies of tactile sensors is given in Table 1.

Touch sensing comprises detection and measurement of the contact point force. Meanwhile, tactile sensing involves not only the detection and/or measurement of the spatial distribution of forces perpendicular to an area but also the interpretation of the resultant information. Accordingly, tactile sensing entails a coordinated group of touch sensors. Slip sensing encompasses the detection and the measurement of the movement of an object relative to the sensor [25]. Principles of sensor types are listed in Table 1 and they have been reviewed in a recent work by Saccomandi et al. [26]. Briefly, piezoresistive sensors measure changes in the resistance of a contact when force is applied. Piezoresistive sensors are normally fabricated in conductive rubber or made with piezoresistive inks applied in specific patterns. A maximum resistance value is registered when no contact or stress is applied to the sensor. Conversely, the resistance decreases with increasing pressure or stress at the contact point. Piezoresistive sensors have a wide dynamic range, durability, good overload tolerance, low cost and ability for fabrication in very small sizes. However, they suffer from limited spatial resolution, the challenge of individually wiring multiple sensor elements, susceptibility to drift and hysteresis [27]. Tactile sensors operating by capacitive transduction measuring the variations of capacitance from an applied load over a parallel plate capacitor. The capacitance is related to the separation and area of the parallel plate capacitor, which uses an elastomeric separator to provide compliance [28]. Although capacitive sensors are susceptible to external fields, they have been widely implemented for the development of “taxels” that mimic aspects of mechanoreception in human fingers. Capacitive sensors can be fabricated for small size applications, allowing their construction and integration into dense arrays in compact spaces, e.g., palms and fingertips [29]. They generally feature high sensitivity; long-term drift stability, low temperature sensitivity, low power consumption and sensing of normal or tangential forces. Limitations include significant hysteresis.

Optical sensors function by transducing mechanical contact, pressure or directional movement, into changes in light intensity or refractive index, which are then identified using state-of-the-art vision sensors [30]. A potential disadvantage is that the sensors should be equipped with light emitters and detectors (e.g., CCD arrays), leading to increased bulk. However, optical sensors are unique due to their potential for high-spatial resolution, immune to electrical interference, lightweight and do not require complex wiring that is commonly encountered in capacitive and piezoresistive sensors [31]. This has led to the integration of optical tactile sensors into various robotic systems [32]. Magnetic tactile sensors are designed to detect changes in magnetic flux, induced by an applied force based on the Hall Effect leading to magnetoresistive or magnetoelastic sensors [33]. Hall Effect sensors function by measuring differences in the voltage that is generated by an electric current passing through a conductive material submerged in a magnetic field [34]. Magnetoresistive and magnetoelastic sensors can also detect variations in magnetic fields generated by the application of mechanical stress. Advantages of magnetic sensors are their high sensitivity, wide dynamic range, very low hysteresis, linear response and general robustness. However, they are susceptible to magnetic interference and noise. Applications can be hindered by the physical size of the sensing device, and by the need to operate in nonmagnetic environments.

**Figure 4:**
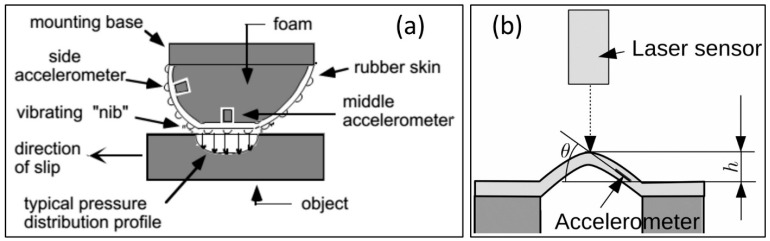
(**a**) Structure of the accelerometer-based artificial fingertip that detects incipient slip. The two accelerometers are used to detect slip-induced vibrations. The foam helps the fingertip conform to the grasped object surface to provide better grip, and reduces grip force control instability problems. The skin is covered with “nibs” that form local contact regions that can slip independently from one another and produce small vibrations. Reprinted/adapted with permission from Ref. [39]. Copyright 2018, IEEE. (**b**) Schematic drawing of experimental setup for measuring height of polymeric skin. Reprinted/adapted with permission from Ref. [40]. Copyright 2019, IEEE.

Piezoelectric sensors produce an electric charge proportional to an applied force, pressure or deformation [35]. Dynamic measurements are challenging and susceptibility to temperature changes makes this sensing technology more convoluted to ensure reproducibility. However, they are appropriate for measurement of vibrations and widely used due to their sensitivity, high frequency response and availability in various forms, e.g., plastics, crystals and ceramics [36,37]. Finally, skin acceleration tactile sensors aim to duplicate the human fingertips that can learn about surface texture and frictional properties. Our fingertips contain cutaneous sensors that are specifically designed to measure contact slip. We as humans can detect displacement of fingertip skin as small as 1–2 microns and a rapid transient displacement occurs. In other words, we sense accelerations as small as 2.5 m^2^/s. These tactile sensors should be carefully constructed from rubbery materials with special geometry and softness–hardness values in order to track object surface texture and frictional properties [38]. An accelerometer is generally embedded in the rubbery constructs as shown in Figure 4. It is still challenging to construct skin accelerator tactile sensors for large areas with high surface coverage density. In addition, integration of polymeric skin motion with other sensors, such as lasers, makes these types of sensors able to be integrated in large numbers.

### 1.3. Analysis and Design Principles of MEMS Devices

**Figure 5:**
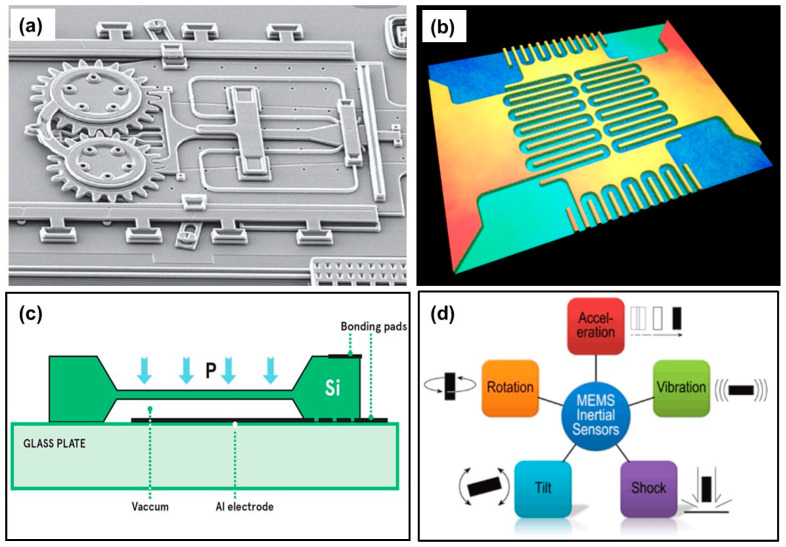
(**a**) A microscopic clutch manufactured from MEMS components. Reprinted/adapted with permission from Ref. [49]. Copyright 2018, The Society of Petroleum Engineers (SPE). (**b**) 3D topography of a MEMs device. (**c**) A cross section schematic of a MEMS capacitive pressure sensor and (**d**) Application areas of MEMS internal sensors.

Micro-electromechanical systems (MEMS) is a process technology that manufactures tiny integrated devices or systems that combine mechanical and electrical components [41,42,43]. An example of such a device is shown in Figure 5a, in which a micron scale clutch, that is a mechanical device that engages and disengages power transmission, is shown. They are fabricated using integrated circuit (IC) batch processing techniques and can range in size from a few micrometers to millimeters (see Figure 5b). The original technology of MEMS devices was based on etching and patterning silicon wafers and surfaces, such as the one shown in Figure 5b. Some performance-relevant parameters of pressure sensors cannot be measured electrically, which is why other techniques are utilized. One such technique is the 3D topography measurement of the thin and sensitive pressure sensor membrane, including membrane thickness measurement to ensure defect-free device and curvature of the pressure sensor membrane at different applied pressures. This avoids unwanted stress and the resistors applied to the pressure sensor membrane can be measured as well to confirm and optimize the positioning and attachment.

MEMS have the ability to sense, control and actuate on the micro scale, and produce effects on the macro scale [44]. In the most classical sense, MEMS is a chip-based technology in which sensors are composed of a suspended mass between a pair of capacitive plates. When the sensor is slanted, a difference in electrical potential is created by this suspended mass. The created difference is then measured as a change in capacitance [45]. MEMS technology sensors are low-cost, high-precision inertial sensors that can be used to serve a wide variety of applications [46,47,48]. Most industries in which MEMS sensors are used operate in extreme temperatures and some MEMS can be sealed to be submerged into shallow water for temporary periods, allowing them to monitor the offshore and subsea pitch and roll applications [49]. MEMS sensors are also resistant to shock and vibration. The origins of MEMS devices go back to the 1950s [50]. Table 2 shows accomplished milestones from the inception of MEMS technologies to the early 2000s, and today, a number of commercialization breakthroughs took place [50].

A typical MEMs pressure sensor can be described as follows: Two of the most common ones are piezoresistive and capacitive. In both cases, a flexible layer is created which acts as a diaphragm that deflects under pressure but different methods are used to measure the displacement, as shown in Figure 5c. To create a capacitive sensor, conducting layers are deposited on the diaphragm and the bottom of a cavity to create a capacitor. The capacitance is typically a few picofarads. Deformation of the diaphragm changes the spacing between the conductors and hence changes the capacitance (see Figure 5c). The change can be measured by including the sensor in a tuned circuit, which changes its frequency with changing pressure.

The sensor can be used with electronic components on the chip to create an oscillator, which generates the output signal. Because of the difficulty of fabricating large inductances on silicon, this will usually be based on an RC circuit. This approach is well suited for wireless readout because it generates a high-frequency signal that can be detected with a suitable external antenna. Alternatively, the capacitance can be measured more directly by measuring the time taken to charge the capacitor from a current source. This can be compared with a reference capacitor to account for manufacturing tolerance and to reduce thermal effects. In both cases, the proximity of the electronics and the sensor element minimizes errors caused by stray capacitance and noise. Materials selection for constructing pressure-sensing MEMS is very challenging [51,52,53]; however, certain practices are being established to produce efficient sensors [12,13]. Another technologically important use of MEMS is internal sensors. For instance, MEMS accelerator sensors can be inserted in rotating machine parts where they are exposed to different forces, such as rotation and vibration, tilt, as shown in Figure 5d [54]. In addition, the recently available miniature barometric sensor chips, which include MEMS pressure sensors, can be integrated with printed circuit boards and other electronic interfaces to function as internal MEMS tactile sensor arrays all for as little as US$1 per sensor [55].

## 2. MEMS-Based Barometers and Their Recent Applications in Tactile Sensing

Many of today’s modern barometers utilize MEMS technology, making them capable of measuring pressure in a more compact and flexible structure. This allows them to be used in smaller applications such as mobile devices and watches. A MEMS barometric pressure sensor detects atmospheric pressure based on how it affects its diaphragm. Simply speaking, the more the diaphragm deforms, the higher the pressure. The air pressure can be monitored based on the piezo-resistive effect. With the mechanical stress of the diaphragm generated by air relative to a reference pressure cavity cell under the diaphragm, the barometric pressure can be evaluated. These types of sensors are quite popular in the portable weather stations where barometric pressure resolution in mBar is enough to meet the requirements [56]. Nevertheless, these types of sensors have nonlinear temperature responses and require calibration that is more complex. In addition, due to their high pressure-noise level, they cannot cover all applications that require low-pressure noise, fast transient response and temperature stability and low power consumption.

**Figure 6:**
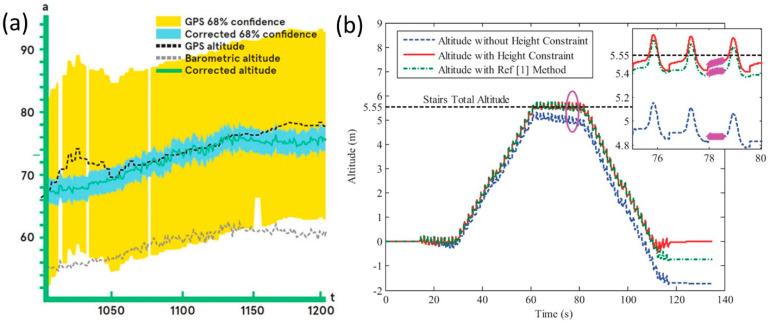
(**a**) Confidence bounds or GPS, barometric and fused altitude measurements. Reprinted/adapted with permission from Ref. [57]. Copyright 2014, IEEE. (**b**) Altitude curves calculated by different methods/algorithms from a pedestrian navigation-based MEMS pressure sensor and its error correction algorithm. Reprinted/adapted with permission from Ref. [58]. Copyright 2017, Emerald Publishing.

Capacitive MEMS technology offers excellent pressure noise, very good pressure accuracy and low power consumption. MEMS-based barometers have been also embedded in smartphones and wearable devices, leading to extensive new applications. For instance, MEMS-based pressure sensors can be used in conjunction with other sensors to track and recognize a wide range of human activity including altitude change recording [57]. However, these MEMs devices should be carefully designed and constructed with high accuracy, and they need to be tested against other standards, such as satellite GPS data, as shown in Figure 6a. In the figure, a MEMS-GPS pressure sensor algorithm was tested using several commercial cell phones (GPS) while hiking, bicycling and riding in a car in both urban and mountainous environments in and around the San Francisco Bay Area [57]. GPS altitude measurements are shown in dashed black and barometric in dotted green. A 68% confidence bounds of GPS measurements, as reported by a sensor, is shown in yellow. The altitude corrected by the algorithm is shown in blue, along with an estimated 68% confidence bounds, shown in pink in Figure 6a. In Figure 6b, exemplifies an optimized algorithm result for low-cost pedestrian navigation MEMS system (PNS) to correct the heading drift and altitude errors, thus achieving high-precise pedestrian location in both two-dimensional (2-D) and three-dimensional (3-D) space [58].

Recent technological progress also enabled the fabrication of low-cost, robust force–torque sensors using MEMS barometer chips. MEMS barometers can be modified to serve as tactile sensors with exceptional sensitivity (<0.01 N), linearity (<1%) and bandwidth (>100 Hz); moreover, they are mass-produced for consumer applications at minimal cost and maximal performance. By casting them in rubbers and positioning them in appropriate configurations, they can be rehabilitated to measure forces and torques [59,60]. As mentioned earlier, the ability to recognize object slip via tactile feedback facilitates humans to accomplish complex manipulation tasks including sustainment of a stable grasp. Despite the convenience of tactile data for many applications, tactile sensors have yet to be extensively used in industrial robotics surroundings; part of the challenge lies in identifying slip and other events from the tactile data torrent. Learning-based methods to detect slip using barometric MEMS tactile sensors have been recently presented [61]. These sensors have favorable properties including high durability and reliability, and are cost effective. It has been argued that barometric tactile sensing technology, combined with data-driven learning, can be suitable for many manipulation tasks such as slip compensation [61].

**Figure 7:**
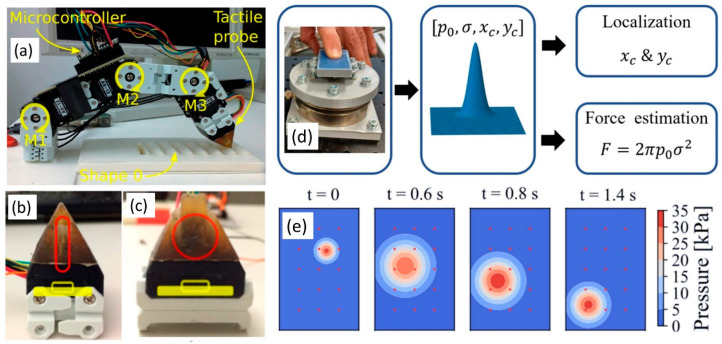
Experimental setup: (**a**) Robot finger composed of three motors: M1 is the “bottom” motor; M2 is the “middle” motor; and M3 is the “top” motor. The microcontroller is attached on top of Motors M1 and M2. (**b**) Front view of the tactile probe: the MARG system is embedded under the red circle; the pressure sensor is under the yellow overlay in the black 3D printed collar. (**c**) Side view of the tactile probe. Reprinted/adapted with permission from Ref. [62]. Copyright 2017, MDPI. (**d**) Illustration of the working principle of the model-based simultaneous localization and force estimation. Left: Finger creates contact with the sensor. Middle: Optimization finds parameter values of the parameterized Gaussian pressure distribution that best fits the pressure data. Right: The found parameters values are used to simultaneously locate the contact and estimate forces. Reprinted/adapted with permission from Ref. [63]. Copyright 2022, IEEE. (**e**) Reconstructed pressure distribution during the slip experiment on the tactile sensor. Reprinted/adapted with permission from Ref. [62]. Copyright 2017, MDPI.

Another unique example is tactile profile classification using a multimodal MEMS-based sensing module [62]. A sliding motion was performed by a robot finger (i.e., kinematic sequence of three motors) carrying the tactile probe on the tip. The probe was made up of magnetic, angular rate and gravity sensors (MARG) and a deep MEMS barometer sensor, embedded in a flexible compliant construction. When the tip was rubbed over a surface (see Figure 7a–c), the MARG unit vibrated and the deep pressure sensor captured the overall normal forces exerted. The tactile probe was used to collect data over seven synthetic shapes (profiles) having an algorithm in frequency and time domain, designed with multiscale principal components analysis and a multilayer neural network [62]. The achieved classification accuracies of 85.1% to 98.9% for the various sensor types demonstrate the usefulness of traditional MEMS as tactile sensors embedded into flexible substrates.

In a similar study, a soft barometric tactile MEMS sensor was developed to simultaneously localize contact and estimate normal force with validation to detect slip in a robotic gripper [63]. The MEMS-based barometric sensor array was covered with an elastomer layer (see Figure 7a–c), with the sensor signals being interpreted in real-time on the basis of a parameterized Gaussian type of distribution. The contact location was determined by finding in real-time the matching parameters of the Gaussian distribution that on its turn is used for normal contact force approximation (see Figure 7d). Their experimental results indicated accuracies in terms of localization of 0.5 mm and normal force errors of 10% in force ranges up to 25 N and 15% in high force ranges of 25–50 N. The MEMS-based sensor arrays were able to detect slip when gripping various objects [63]. Figure 7e shows the varying pressure distribution during the slip experiments, illustrating the movement of the estimated Gaussian pressure distribution that was used to identify slip.

**Figure 8:**
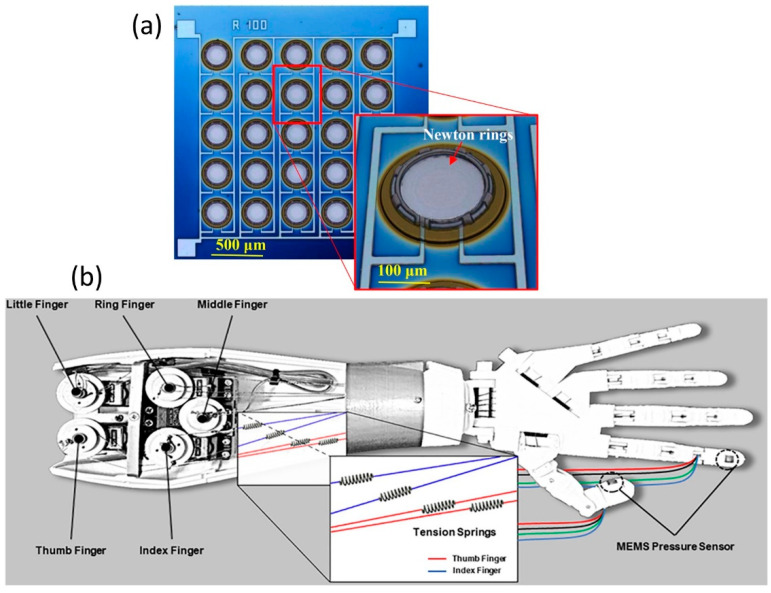
(**a**) Microscopic image of MEMS pressure sensor. Inset: 3D image of a diaphragm with interference patterns (Newton rings), indicating complete sealing. Reprinted/adapted with permission from Ref. [66]. Copyright 2015, IEEE. (**b**) A 3D printed robotic hand with actuation mechanisms. Small capacitive MEMS pressure sensors shown in (**a**) are ideal for the fingertips of the hand. Reprinted/adapted with permission from Ref. [67]. Copyright 2019, SAGE Publishing.

It is important to note that the performance of the multi-dimensional force sensors is largely dependent upon the mechanical assembly of elastic body. Additionally, the calibration process of the pressure sensors needs to be analyzed carefully, and problems in calibration should be eliminated. Interdimensional coupling error is one of the central factors affecting the measurement precision of the multi-dimensional pressure sensors. Thus, reducing or even removing dimensional coupling error becomes an essential requirement in the design of multi-dimensional force/pressure sensors, and the decoupling techniques of the multi-dimensional force sensors must be advanced more [64]. Kõiva et al. [65] produced a tactile sensor for the Shadow Dexterous Hand’s palm. They improved the tactile sensor features by utilizing state-of-the-art barometer-based tactile sensing with linear (R^2^ ≥ 0.9996) sensor output and no perceptible hysteresis. Implementing a revised neural network architecture further improved the average classification accuracy to 96% in a 5-fold cross-validation. They designed experiments to estimate the stiffness of different objects with considerable performance boost in estimation accuracy compared to their earlier Shadow Dexterous Hand [65]. Nguyen et al. [66] constructed a MEMS-based capacitive pressure sensor with pre-stressed sensing diaphragms for attaining a linear response with applied pressure. The sensor was operated in touch-mode by using sensing pressure diaphragms with compressive residual stress over insulated counter electrodes. Finite Element Method (FEM) modeling showed that a sensing diaphragm with residual stress could deliver a better linear response than a stress-free diaphragm. The MEMS sensor was fabricated on a Si substrate using surface micromachining and low-pressure chemical vapor deposition sealed the pressure cavity and formed a dielectric insulation layer (see Figure 8a for a schematic description). The MEMS pressure sensor responded linearly in the pressure range of 16–215 psi with a sensitivity of 0.092 pF/psi and full-scale nonlinearity of 3.3% without compensation [66]. This sensor array (about 16 sensors/mm^2^) can be integrated into robotic hands like the one shown in Figure 8b, which was fabricated by 3D printing.

Moreover, virtual reality (VR) becomes an effective tool capable of visualizing complex systems in full details and with a high level of interactivity with small pressure sensor assays to help with shape recognition and grasping [68].

## 3. Flexible MEMS-Based Tactile Sensors

### 3.1. A Brief Review of Principles and Applications of Flexible Capacitor, Piezoelectric, Magnetic and Conductive Pressure/Force Sensors

**Figure 9:**
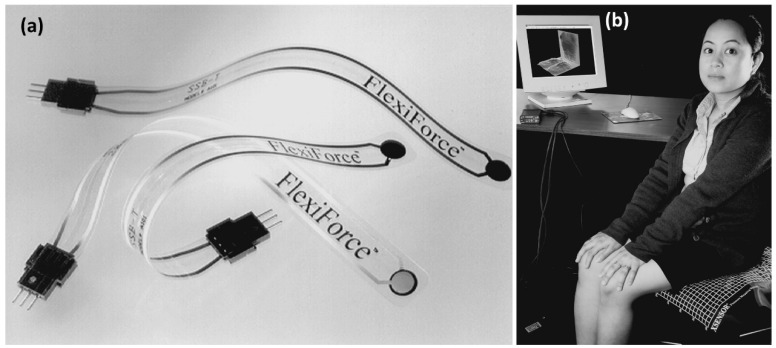
(**a**) Flexible commercial force sensors with a single sensing element are shown having a circular and located at the tip of the flexible plastic strip and (**b**) pressure mapping maps placed on chair sitting face and the back side having the integrated array of sensors, which are linked to a small electronics interface box that is connected to a standard computer. The computer screen shows a real-time image of the pressure distribution. Reprinted/adapted with permission from Ref. [70]. Copyright 2002, Emerald Publishing.

A recent review by Claver and Zhao [69] presents the cutting-edge progress of e-skin-based (electronic skin) flexible pressure sensors, such as piezoresistivity, capacitance, triboelectricity and piezoelectricity covering very recent works in last five years. Therein, they reviewed working principles, structure design, materials utilized and performance of numerous flexible pressure sensors. Their work did not specifically identify and classify flexible pressure sensors in the form of MEMS. Ashruf [70] presented an earlier (early 2000s) overview on the practices for the measurement of interface pressure or force between (soft) objects and reviewed uses of single sensor elements as well as integrated arrays of sensors to obtain pressure maps. In many applications, the flexible pressure/force sensors merely distinguish two states, i.e., on or off. The demands on the performance of these sensors are generally lower than that of the analogue sensors. Examples of analogue sensors are robotic touch and picking sensors and load cells. An example of an analogue sensor is shown in Figure 9a. In the figure, a flexible commercial force sensor with a single sensing element is shown having a circular and located at the tip of the flexible plastic strip. An example of a complete pressure mapping system, having the integrated array of sensors, which is linked to a small electronics interface box shown in Figure 9b. The interface box is connected to a standard computer. The computer screen shows a real-time image of the pressure distribution due to a person sitting in a chair (Figure 9b). Integrating sensors onto a flexible substrate will add functionality to flexible and moving machines. So far, several attempts have been made to mount MEMS sensors on flexible substrates, while more work will still be needed to fabricate sensors integrally on the substrate. For instance, manufacturing MEMS sensors on flexible substrates along with display elements will offer new sensing aptitudes and enhanced functionality. Such an attempt was demonstrated by Lakamraju et al. [71] in which MEMS capacitive sensors were fabricated in a low temperature, flexible amorphous silicon process. Their sensor was used for acoustic detection with potential applications in blast dosimetry [71].

As can be seen in Table 3, flexible MEMS pressure sensors have not become conventional yet compared to other flexible pressure sensors based on silicone rubber or paper-based technologies [72]. Table 4 shows typical applications for flexible pressure sensing arrays and their corresponding pressure ranges.

Chitra et al. [83] constructed a comb drive-based MEMs capacitive pressure sensor for measuring the pressure inside the lubricating system. MEMS technology utilized by the authors allowed the diaphragm to be very thin compared to conventional machining. The comb drive capacitive pressure sensor separated the pressure-sensing diaphragm from its capacitance sensing movable comb plate by a mechanical coupling that increased the pressure sensitivity. The MEMS sensor worked efficiently in the range of 30 °C to 270 °C with atto (10^−18^) Farad sensitivity. However, such sensitive MEMS devices need to be fitted to flexible compliant platforms. One of the most commonly employed soft polymers in capacitive flexible pressure sensor technology is silicone or polydimethylsiloxane (PDMS) and a comparison of various capacitive pressure sensors based on PDMS microstructure patterns is given in Table 5 with reported sensitivity and detection limits.

**Figure 10:**
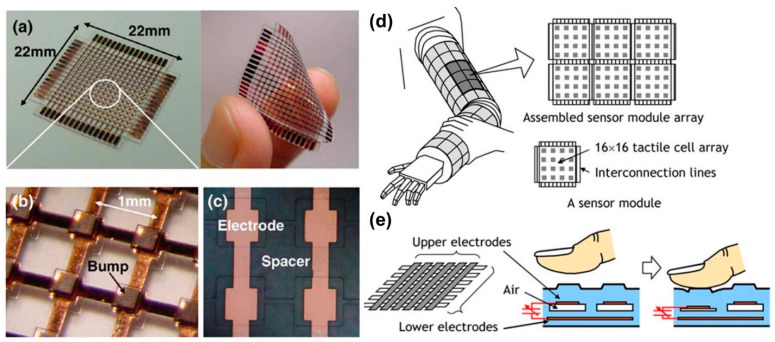
(**a**) Fabricated tactile sensor module, (**b**) magnified view of cells and (**c**) the bottom electrodes and spacer layer of four tactile cells. Schematic diagram of the proposed modular expandable tactile sensor: (**d**) sensor module array and (**e**) structure of single tactile cell. The tactile cell capacitance changes as the air gap is squeezed according to applied force. Reprinted/adapted with permission from Ref. [74]. Copyright 2006, IEEE.

Lee et al. [74] fabricated a modular and flexible expandable capacitive tactile sensor using PDMS rubber. The sensor module comprised 16 × 16 tactile cells with 1 mm spatial resolution, comparable to human skin, and interconnection lines for expandability. They fabricated the sensors by bonding five PDMS layers together forming a cell size of 600 × 600 m^2^ with a single cell capacitance of 180 fF. The flexible MEMS-based tactile sensors demonstrated a sensitivity of 3%/ mN within the full-scale range of 40 mN (250 kPa). Photographs of the fabricated cells are shown in Figure 10a–e schematically demonstrate robotic arm layout of the sensor pads and the capacitive working principle of a typical cell with embedded top and bottom electrodes [74].

A unique review article on flexible MEMS sensors focused on nitride-based materials for tactile and flow sensing in robotics [89]. Therein, the authors compiled works based on aluminum nitride and silicon nitride MEMS by exploiting the material stress differences among the constituent layers of nitride-based (AlN/Mo, SixNy/Si and AlN/polyimide) mechanical elements to produce microstructures, such as upwardly-bent cantilever beams and bowed circular membranes. These MEMS utilize piezoresistive properties of nichrome strain gauges and direct piezoelectric properties of aluminum nitride (AlN) towards mechanical strain/stress detection [89]. A recent work reported on the fabrication of a polyvinylidene fluoride (PVDF)/fullerene-based polymer piezoelectric sensor array for the detection of pulmonary pressure under blowing conditions that did not use any silicon-based MEMS [90,91]. The MEMS flexible sensor was manufactured by coupling the MEMS techniques with nanomaterials. Firstly, the MEMS-based Wheatstone bridge model with piezo electric structure was made by writing of nanowires via a high viscosity-induced nanoscale diffusive layer [90]. Afterwards, the authors prepared the PVDF/Fullerene-based nanofiber network and coated over the Wheatstone bridge for pressure sensor application, further the sensors were optimized for the pulmonary pressure measurement. Dahiya et al. [1] classified flexible MEMS as “MEMS on plastic” based on the highly cited work by Engel et al. [92] on polymer micromachined multimodal tactile sensors. In fact, the flexible MEMS sensors developed by Engel et al. [92] were tailored to sense the hardness, thermal conductivity, temperature and surface contour of a contact object for comprehensive evaluation of contact objects and events. Among them, pressure is an input in sensing the hardness of an object. Following that work, surface textures were also characterized by using polymer-based microelectromechanical systems (MEMS) tactile sensor array and statistical computation [93]. Texture classification was achieved by using a maximum likelihood decision rule that optimally categorized patterns in the presence of noisy signal to manage texture variation and random noise. Using a 4 × 4 sensor array, the authors identified a variety of simple textures even though they used low-sensitivity mechanical strain gauges serving as a transduction elements. This pioneering work resulted in acceptable overall performance of 68% correct classification. The authors also presented future work to improve identification performance of the system [93,94].

Takao et al. [95] designed and used a new concept of silicon multi-functional tactile imager targeting 3-D object shape sensing. They attempted to eliminate problems related to mechanical stroke of the small sensor pixels that are usually very short and limited due to the existence of hard silicon substrate under them. This problem is a main limitation of monolithic silicon tactile image sensors based on MEMS. To this end, a large and air-pressurized single silicon diaphragm was used as a mechanically soft sensing structure, with a tactile sensing array as flexible integrated circuits placed over it. They also integrated signal-processing circuitry for the sensing array around the diaphragm. The sensing diaphragm was swollen pneumatically by an air-pressure to obtain a soft and flexible surface. Contact force distribution of touching objects was perceived from stress distribution on the deformed diaphragm. In addition, hardness distribution was extracted by ’amplitude-modulation’ of the diaphragm vibration.

**Figure 11:**
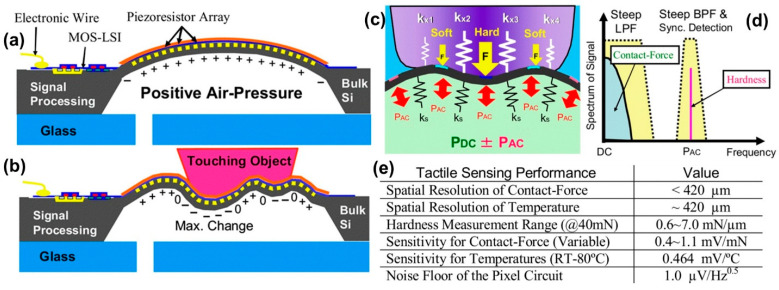
Cross-section and surface stress of the tactile imager, (**a**) before object contact, (**b**) after object contact. (**c**) Detection principle of distributed hardness using pressure vibration; (**c**) Repulsive force distribution for ± PAC depending on local hardness, (**d**) Power spectrums of piezoresistor signal. Contact-force signal appears as the average around DC, while hardness signal appears at PAC. (**e**) Summary of measured performances (10 μm-thick sensing diaphragm). Reprinted/adapted with permission from Ref. [95]. Copyright 2007, IEEE.

Figure 11a,b display the cross sectional image of the MEMS tactile sensor. When nothing touches the swollen diaphragm, the diaphragm surface is swollen to upwards, and tensile membrane stress is applied on the 2-D piezoresistor array, as shown in Figure 11a. Output voltage from each piezoresistor pixel in this situation is measured as the ‘initial state’. When it makes contact with an object, the swollen diaphragm is deformed compliantly, as shown in Figure 11b. The change in stress distribution from the initial state becomes maximum at the tip of the object and by reading the distribution of stress change over the sensing array, the shape of the object can be distinguished as a contact-force image with a signal band around DC in the frequency domain. Figure 11c,d elucidate the detection principle of hardness distribution (k values depicted as resistance). When an object touches the sensor surface, a vibrating force with small amplitude is applied to the object since overall surface of the diaphragm vibrates at a small amplitude of ∼0.1 μm by ± PAC (alternating pressure component). The repulsive force from the object depends on the local hardness of the object as k × 1–k × 4, shown in Figure 11c. If the pressure is vibrating at the frequency of PAC, the repulsive force on the object will also vibrate, and the output voltage converts to an alternative signal. Thus, power spectrum of a piezoresistor signal contains a contact-force signal in the signal band and a hardness signal at PAC as shown in Figure 11d. Since the repulsive force increases with hardness for a constant displacement, the higher the item hardness, the larger the output amplitude. The performances of the tactile imagers with flexible deformation of sensing pixel arrays reported by the authors are summarized in Figure 11e.

### 3.2. MEMS Tactile Sensors Utilizing Triboelectric Effect

Next, we will discuss triboelectric-based MEMS tactile sensing systems and technologies for flexible platforms. The triboelectric signal relates strongly to the specific charge condition of the surface material of a target object, in conjunction with specific transducers such as an electromagnetic inductance transducer, in which the inductive signal reveals the electromagnetic characteristics at a certain depth inside the object. Introducing machine learning algorithms to the sensor arrays, for instance, the triboelectric signals and inductive signals, can be exploited for object identification. Triboelectric nanogenerator (TENG) exploits contact electrification and electrostatic induction mechanisms to produce electrical signals in response to a mechanical deformation. Consequently, friction and/or surface contact between the functional layers of the triboelectric device is quite critical during operation. TENGs can be developed using a wide range of materials with dissimilar charge affinities. A higher gap between the charge affinities among active layers within the device will result in a stronger triboelectric response. A notable work by Wang et al. [96] developed flexible and wearable PDMS-based triboelectric nanogenerator for self-powered tactile sensing. Therein, the authors used MEMS manufacturing process to ensure good sensitivity and high output performance to the sensor. Their triboelectric MEMS sensor directly converted mechanical energy into electric energy and could light up 110 green Light-Emitting Diodes (LEDs). The sensor displays demonstrated good sensitivity (2.54 V/kPa), linearity (R^2^ = 0.99522) and stability (over 30,000 cycles). The authors argued that their tactile sensors can be conformably attached to human skin to monitor joint movements, as wearable tactile devices. A very recent and significant work by Li et al. [97] describes a triboelectric-inductive hybrid tactile sensor for highly accurate object recognition. They fabricated a robotic gripper with random operation settings that could identify eight different fruits with an accuracy as high as 98.75%. Additionally, the hybrid sensor could recognize objects packaged in different ways.

**Figure 12:**
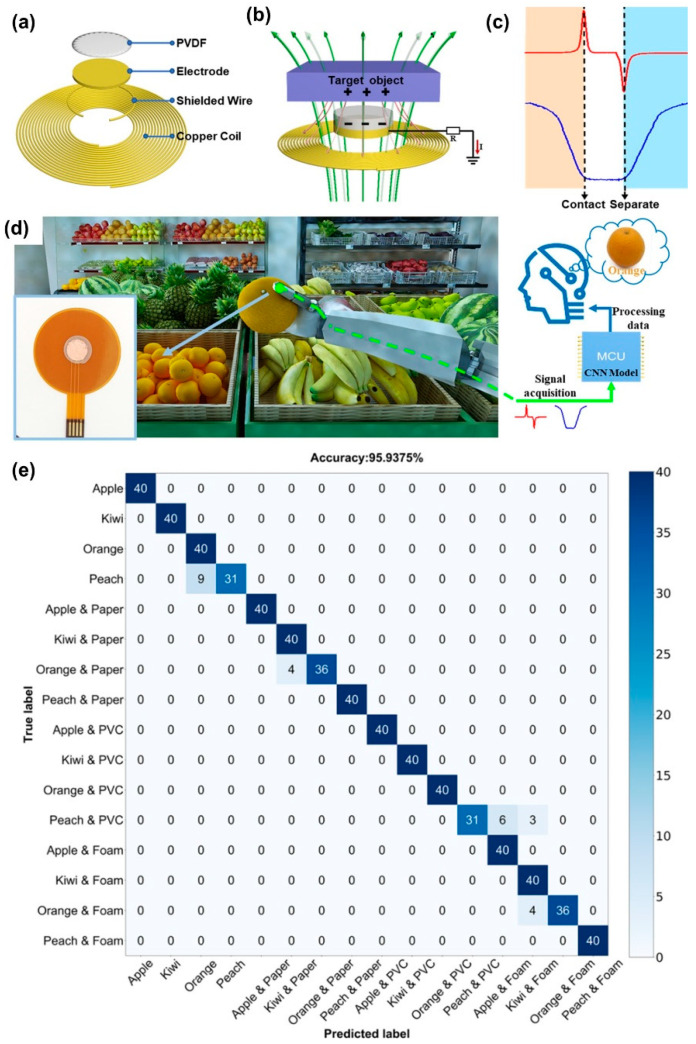
Schematic illustration of the dual-mode tactile sensor. (**a**) Design of the dual-mode tactile sensor. (**b**) Working mechanism of the dual-mode tactile sensor. (**c**) Typical signals from the dual-mode tactile sensor when touching an object. (**d**) Concept of object recognition by a robotic gripper equipped with the dual-mode tactile sensor. (**e**) The confusion matrix derived from the CNN model with the database of dual-mode signals. Reprinted/adapted with permission from Ref. [97]. Copyright 2022, Elsevier.

They reported recognition accuracy of four different fruits in three different packages as high as 95.93%. Integrating neural networks and deep learning with tactile sensors can produce better haptic perception and more accurate object recognition. The method has also been proven effective in solving classification problems without relating to an explicit model. Machine learning is an active methodology in dealing with classification problems with complex input signals. It can extract characteristic features from seemingly unrelated data sets. They implemented a one-dimensional convolutional neural network (CNN) for the dual-mode signal processing and data analysis. A schematic representation of the hybrid sensor is shown in Figure 12a in which the hybrid sensor contains two units. A single-electrode triboelectric sensor composed of a PVDF triboelectric layer and a copper electrode read the surface charge interaction when in contact with a surface. A planar inductance sensor, having constantan coils, measures the electromagnetic induction of the object. These two sensors are organized in concentric circles and assembled in the same plane to ensure the synchronization of the two signals. A grounded shield ring is also used to avoid mutual interference.

The working mechanisms of the triboelectric sensor are given in Figure 12b. When external stimuli occur by triboelectrification or electrostatic induction, a positive voltage is generated between the electrodes, as shown in Figure 12c. Similarly, when the stimuli are removed, the electrical potential will revert to its original value, generating a negative voltage. The PVDF film is laser-engraved to mimic the surface texture of a human finger to improve the amplitude of the triboelectrification. The working mechanisms of the inductance sensor are also shown in Figure 12b. Alternating current flowing through the concentric coils can generate alternating electromagnetic field, which will induce an internal current loop inside the object within the coils, causing mutual inductance. The intensity of mutual inductance will increase as the inductance coil approaches an object, reaching a maximum value when contact is made (Figure 12c). Figure 12d shows a photograph of a robotic hand fitted with the hybrid sensors to recognize a variety of fruits. The authors chose four kinds of fruits wrapped by paper bags, plastic bag, foam and none to form 16 test samples. Figure 12e shows a comprehensive machine learning process from signal acquirement to data processing. Data from 200 object-gripping tests with random grasp settings were collected to construct a new training set. The typical signals from the dual-mode sensor were analyzed and after training the CNN model, the confusion matrix exhibited a high recognition accuracy of 95.93% (see Figure 12e), indicating that the fruits inside different packages were effectively identified.

## 4. Hardness/Stiffness Sensing by MEMS Devices

In the MEMS field, the detection capability for hardness has been realized in various devices but the concept of hardness sensing under a controlled contact force remains to this day difficult to apply to the cases of unstable contact force. On the other hand, hardness sensors, having a compensation function of contact force with other elements, have been demonstrated to eradicate unstable contact force manipulations. Hence, MEMS hardness sensors, having a single element and a low dependence on contact force, are in high demand [98]. Zhao et al. [99] fabricated a tactile sensor to test the hardness of soft tissues motivated by robot–human interactions. The MEMS-based sensor was made up of a tandem spring design fabricated by a direct silicon-to-PCB fabrication/packaging scheme. The authors studied the sensitivity of the tactile sensor and found a scaling factor dependent behavior on contact conditions including contact angle and contact forces. The MEMS-based hardness sensor was able to operate within a hardness range of 0.3–360 psi (2.1–2482.1 kPa), covering most biological tissue types of interest. Similarly, the work of Peng et al. [100] developed a new sensor composed of an array of MEMS capacitive sensing membranes with different stiffnesses to address the challenges related to estimating tissue stiffness by manipulating the relative capacitive change data from a series of sensors that were micro-fabricated through a five-mask surface micromachining process. Preliminary testing on polymers with different compliances indicated a sensing resolution of 0.2 mN for force sensing and at least 0.2 MPa for stiffness sensing [100].

Micro and nano-mechanical resonators (μ-resonators) signify an important building block in MEMS devices. The quartz crystal microbalance (QCM), for instance, is a simple, cheap, high-resolution resonant-based mass sensing technique that has been used for several decades as a tool to detect mass and sensitive changes in weight. In recent years, the advances in MEMS fabrication technology enabled miniaturizing the conversional QCM sensors from several millimeter to micrometer range utilizing not only piezoelectric but also piezoresistive or electrostatic for actuation and detection. To date, many different μ-resonators have been fabricated. The vast majority of designs are modeled as a one-degree-of-freedom (1 DoF) resonator (z-direction for an out-of-plane flexural mode cantilever) [101]. They were also implemented in pressure sensing MEMS. As an example, Pattnaik et al. [102] constructed a novel optical MEMS pressure sensor using integrated optical ring resonator over a micro-machined silicon circular diaphragm targeting 300 kPa range. As the diaphragm deflected due to the applied pressure, stress induced refractive index change in the waveguide led to changes in the phases of the light propagating through resonator. Such a shift in the resonance frequency due to this phase change can be correlated with the applied pressure. The phase response of the sensor was about 19 /spl mu/rad/Pa for 1 mm radius 65 /spl mu/m thick circular diaphragm. The wavelength shift of 0.78 pm/kPa was obtained in the sensor and the sensor could be used up to a range of 300 kPa. Since the wavelength of operation is around 1.55 /spl mu/m. Wang et al. [103] reported an all-quartz high accuracy MEMS absolute pressure sensor based on high Q double-ended tuning fork (DETF) resonator (DETF is a low frequency resonating element with beams connected at each end by a base. The main parts of the MEMS pressure sensor, including the DETF resonator, diaphragm and back cavity structure, were made of quartz crystals that were bonded together as a ‘sandwich’ structure to form the absolute pressure sensing covered with a glass paste under low temperature and vacuum condition to eliminate the thermal stress effect. Their experimental results indicated that the quality factor of the DETF resonator was about 61,000, the sensitivity was 7.35 Hz/kPa in the operating range 0–250 kPa, the pressure error was only 0.021% FS over the temperature range −40 °C to +60 °C. Hasan et al. [104] proposed using the onset of nonlinear dynamics to activate an electrically actuated microscale beam as a tunable-threshold pressure sensor. The process was based on a straight microscale beam with a proof mass excited at its subharmonic frequency and a curved microscale beam excited to achieve snap-through (complete buckling about its neutral axis) at the threshold pressure. In other words, the straight-beam MEMS resonator operated at twice its natural frequency with a linear curve within the range of operation and a MEMS arch-beam operated around its primary resonance having a logarithmic curve. In both cases, the onset of the nonlinear response was used as a digital signal as these nonlinear behaviors were characterized by a sudden change in the response. For sensing with m-resonators implementing piezoelectric principles, the work by Lu et al. [105] can be consulted.

The mechanical quality factor (Q) of a MEMS resonator is a very important parameter and property and is defined as a measure of the energy decay rate in each cycle of vibrations. The higher the resonator Q, the longer that coherent energy will be sustained prior to leaking into the environment. The Q is correlated with the thermomechanical displacement noise of a resonator that is crucial for designing tactile sensors with a high signal-to-noise ratio (SNR). In such pressure or tactile sensing applications, an increase in Q of the resonator will improve the thermomechanical SNR [106]. Compared to other resonant devices, quartz resonators are less affected by external environmental factors due to their own high quality factor Q; however, effects such as temperature, vibration and humidity cannot be ignored in high-precision applications, and they need to be compensated. To this end, Zhang et al. [107] fabricated a high sensitivity quartz resonant pressure sensor (quartz double-ended tuning fork, DETF) with differential output and self-correction.

**Figure 13:**
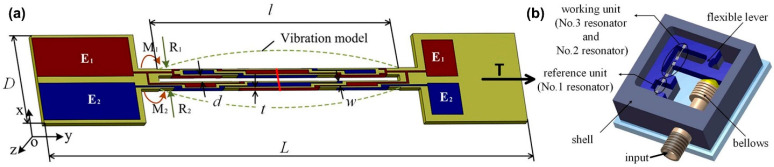
(**a**) Schematic diagram of the structure and electrode arrangement of quartz double-ended tuning fork (DETF). (**b**) The main structure diagram of the sensor. Reprinted/adapted with permission from Ref. [107]. Copyright 2019, AIP Publishing.

The DETF quartz resonator consists of a pair of tines and two mounting bases at both ends as shown in Figure 13a and the structure and the electrode configuration of the DETF are schematically shown in Figure 13b. The tines are oriented along the mechanical axis, y-axis, of the quartz crystal. Electrodes are distributed on the mounting bases and around the surfaces of the tines along the length direction [107]. Three DETF resonators, a titanium alloy elliptical flexible hinge and a tin bronze bellows were connected together to form the sensor, as shown in Figure 13b. Among them, the bellows function to convert the pressure to be measured into displacement, and this displacement is amplified by the flexible lever, and the DETF resonator converts the displacement into a frequency signal. The first resonator is the reference unit and works as a temperature sensor. Its temperature-dependent signal was introduced into the differential frequencies of the other two resonators to counteract the effect of temperature on the sensor. The second and third resonators act as the pressure sensing elements. Several related research studies [108,109] published in recent years are listed in Table 6. The fourth listing in the table is a commercial product (Paroscientific series-1000; Redmond, WA, USA).

An alternative tactile sensing mechanism suitable for assessing stiffness of the objects is based on the theory of the deformation of thin plates [110]. Features of this design include its potentially rugged realization, and its high-accuracy measurement that is more typical of force sensors than of tactile sensors.

Fouly et al. [111] used this concept to model and experimentally test a three-tip configuration tactile sensor for compensating the error due to soft tissue surface irregularities during stiffness detection. They used three springs in order to achieve an output that is independent of the sensor displacement and/or the contact angle. To investigate the performance of the new sensor, a finite element model was developed, using lumped springs; then, a macro-scale sensor was fabricated and tested. Combination of this approach with a stiffness measurement system that exploits the contacting object’s indentation profile dependent on applied forces could be the next state of the art for tactile sensors. The indentation edge (shape, depth and profile) is the vital constraint for determination of the stiffness measurement, because it is the main cause factor for the measurement of the stiffness of an object. To enhance the perception capability of the system, sensitivity of the indentation edge movement depending on pressure could be controlled by regulating three design parameters, the touch module separation, the piston protrusion and the spring constant. Such stiffness measurement devices can be scaled and optimized to be integrated into various robotic probes due to the simplicity in operation and the aptitude to adjust the sensing range to increase the stiffness measurement accuracy [112]. During sensor–object or surface interaction, tactile information must contain the amplitude of contact force, the distributed force information, the degree of hardness for the contact surface and the local discontinuities in the surface hardness [26,113]. Stiffness and hardness of the internal components of the MEMS capacitive sensors are also critical for reading accurate pressures with high sensitivity. Lower stiffness in the form of softened beams in MEMS accelerometers was found to be very advantageous such that when the stiffness of the accelerometer was reduced by 43% with softened beams, the sensitivity was augmented by 72.6% [114]. In fact, AFM probes are fabricated using processes developed for MEMS devices. Due to the flexibility in the design of MEMS force sensors, an extensive range of measurements, degrees of freedom and resolutions can be achieved, offering more versatility compared to AFM cantilevers [115]. An adjustable-stiffness MEMS force sensor was designed, characterized and could be controlled by a high-bandwidth device containing on-chip sensing and actuation mechanisms, enabling open- and closed-loop modalities. Such sensors can detect forces down to 10 nN with a dynamic range of 71.2 dB and a sensing bandwidth of 3.6 kHz [115].

**Figure 14:**
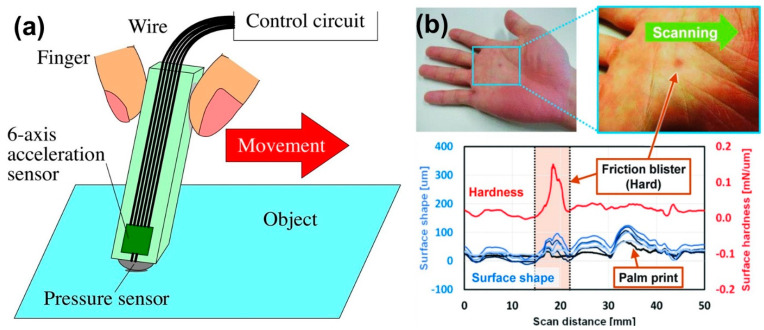
(**a**) Pen type pressure sensor for surface texture and softness detection. (**b**) Measurement results of friction blister on the subject’s palm and comparison of surface shape signal and hardness signal. Reprinted/adapted with permission from Ref. [118]. Copyright 2021, IEEE.

With proper design of array type tactile sensors with differently protruded contactors and a new detection principles simultaneous measurement of high-resolution surface texture and the elasticity (or softness) of the touching object can be accurately detected. For instance, six biaxial detectors for microscale surface roughness and local slip friction were integrated with different protrusion lengths of three levels from the chip edge of sensor [116]. Since indentation depth of the three contactor–sensor pairs depends on softness of object, it was possible to calculate the elasticity of the object via one-way sweeping using the relative relationship between indentation depths. This type of design canceled out the effect of contact angle to the target surface and it can detect different surfaces having elasticity from 0.57 to 2.6MPa [117]. A similar miniature system, by using a convolutional neural network, constructed a pen-type tactile sensor system for roughness recognition of six objects [118]. The average correct recognition rate was about 71% for the experimental data acquired by one user that were categorized into learning data and evaluation data. Furthermore, the total average recognition rate for evaluation data by the other five users for considering each individual using the sensor system was shown to be 42% [118]. Their design is schematically illustrated in Figure 14a. Figure 14b displays the results of measuring a friction blister on the palm of a hand. Friction blisters are traumatic changes in the skin. The surface topography signal measured not only the water swelling of the blister, but also the large unevenness of the palm surface. However, the hardness signal indicated that the friction blister was much harder than the surrounding skin. The hardness signal responded to the friction blister but not to the palm print. The scanner could detect hardness and surface unevenness independently.

In summary, we can indicate that MEMS tactile sensors are categorized by their functions into two types. One class focuses on contact force detection, and to date, most work has been performed in this category. The sensors identify contact force between the sensor surface and the object, by using strain gauges or variable capacitance, etc., as mentioned earlier. This type of sensor is also applied to extract surface morphology information by applying the same measurement principles. The other type, hardness detection, consists of a resonator element that detects hardness of contacted objects using resonance frequency change or multiple spring-like assembly, as summarized in this section. Extracting or measuring multiple contact object properties such as its roughness and its stiffness or harness or even elasticity will lead to high-density and intelligent systems [119,120].

## 5. Textile Integrated MEMS Sensors

Electronic textiles or e-textiles are fabrics that allow electronic components such as batteries, lights, sensors and microcontrollers to be embedded in them. Presently, a majority of MEMS fabrication techniques are predominantly based on the silicon micromachining processes, resulting in rigid and low aspect ratio devices. In fact, fibers or woven or nonwoven textiles made up of special polymers such as polymers with piezoelectric properties can inherently become a MEMS textile with flexible, high-aspect ratio attributes. Vinylidene fluoride-trifluoroethylene-chlorofluoroethylene terpolymer, P(VDF-TrFE-CFE) is one such polymer. The terpolymer exhibits high electrostrictive strain (>7%) with relatively high modulus (>0.3 GPa). It has been also observed that the large electrostrictive strain is nearly constant in the temperature range from 20 to 80 °C. The high room temperature relative dielectric constant (~50), which is the highest among all the known polymers), high induced polarization (~0.05 C/m^2^) and high electric breakdown field (>400 MV/m) lead to very high volume efficiency for the electric energy storage operated under high voltage (~10 J/cm^3^) [121,122]. Note that Electrostriction (cf. magnetostriction) is a property of all electrical non-conductors, or dielectrics that causes them to change their shape under the application of an electric or magnetic field. Once properly constructed, stretchable electrodes, for instance, can be effectively utilized to construct fully flexible tactile pressure sensors for robotic grasping applications [123]. Generally, textiles, silicone polymers or rubbers and graphene have been extensively used for building flexible, stretchable electrodes with strain sensing capabilities [124,125,126,127,128,129,130]. Electrospun nanofiber mats from PVDF and its copolymer have recently been employed as tactile sensors [131], for instance, P(VDF-TrFE) nanofiber mats as 3D pressure sensor demonstrated sensitivity levels at 0.1 Pa. When aligned, the nanofiber mats functioned better as tactile sensors such as the core–shell P(VDF-TrFE) nanofibers having almost 40 times higher sensitivity than pure thin-film PVDF. Table 7 summarizes some technical performance and sensing output of such sensors made with PVDF-based nanofibers having nanoscale additives such as multiwall carbon nanotubes (MWCNTs) and silver. It may be concluded that PVDF and PVDF co-polymer nanofibers represent the most promising materials for flexible tactile-sensing applications.

**Figure 15:**
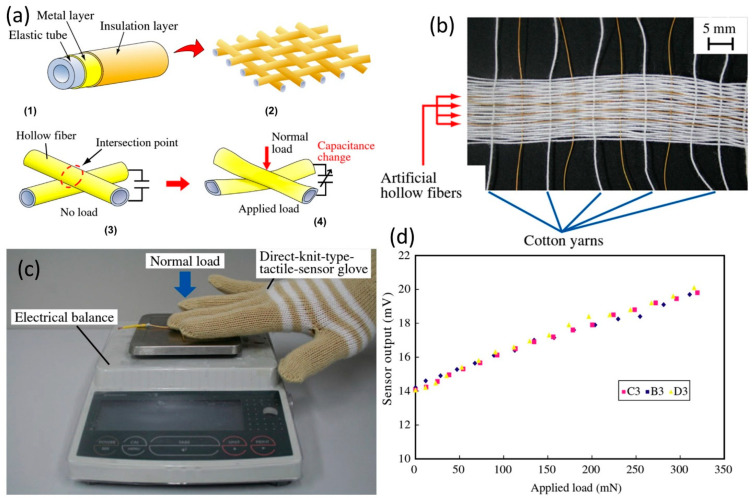
(**a**) Schematic view and operation principle of a fabric tactile sensor: (**1**) artificial hollow fiber, (**2**) fabric tactile sensor, (**3**) and (**4**) operation principle. (**b**) Woven fabric tactile sensor produced by combining artificial hollow fibers and cotton yarns. (**c**) Experimental method of measuring the relationship between the applied normal load and the sensor output using a direct-knit-type-tactile-sensor glove. (**d**) Relationship between the applied normal load and the sensor output using the patched-type-wearable-tactile-sensor glove. Reprinted/adapted with permission from Ref. [154]. Copyright 2008, Institute of Physics (IOP).

Recently, the development of textile-based sensors has been increasingly implemented in producing more human-oriented monitoring devices. The sensing fabrics are manufactured by combining the conductive and non-conductive yarns (or deposited conductive elements) which can be defined as parts of electrode and gauge, respectively. Textile-based MEMS technology can offer a number of advantages such as simple methods for the problem of constructing low-cost MEMS sensors, fabrics are readily available, lightweight and easy to manufacture, without requiring cleanroom facilities, and fabrics or textiles offer compliance and no lengthy training in constructing the MEMS devices is necessary [150,151,152,153]. For instance, an artificial-hollow-fiber structure MEMS was developed and tested as a fabric tactile sensor, as shown in Figure 15a. The hollow fiber was fabricated by uniformly deposited metal and insulation layers on the surface of a soft hollow tube. A rectangular-shaped fabric tactile sensor was assembled by merging artificial hollow fibers and cotton yarns, such as a cloth as seen in Figure 15b. The sensor functioned in such a way that the contact forces were detected by measuring changes in capacitance at all intersection points of the artificial hollow fibers. It could be either applied as a patch on a cloth or integrated into the knit pattern of the cloth or a glove for instance (see Figure 15c) [154]. The relationship between the applied normal load and the sensor output is shown in Figure 15c. The sensor output increased from 14 to 20 mV, about 1.4 times the values recorded under initial conditions, with increase in applied normal load (Figure 15d).

The work of Suzumori et al. [155] was probably among the first noteworthy studies on MEMS sensor embedded fiber networks. Therein the authors have designed a flexible microactuator for use in miniature robots, composed of fiber-reinforced rubber and actuated by an electropneumatic or electrohydraulic system. The microactuators had several degrees of freedom (including pitch, yaw and stretch) suitable for robotic arms, legs or fingers. A recent review by Kaltsas et al. [156] compiled latest advances in functional fabric sensors relevant to robotics applications including visual, body and tactile sensors. Again in a very recent work, Wei et al. [157] implemented multi-robot collaboration using the alignment process of a MEMS clamps array with a fiber/fabric mesh. The fabric integrated MEMS were used to guide and operate robotic arms with high efficiency. Recent developments in advanced textiles have led to the incorporation of a wide variety of MEMS sensing and computational capabilities into textiles without compromising the flexible and wearable characteristics of the textiles such as textile embedded silicon flexible skins made up of arrays of silicon islands integrated with boron-doped strain gauges and metal pads fabricated using micromachining techniques [158,159]. Nowadays fabrication of conductive micro-spring arrays (hair-like structures) as an electrical contact structure directly on fabrics can be achieved with high accuracy. The micro-spring contact array can be used in forming the electrical circuit through a large area of woven textile, and functions as the electrical contact between weft ribbon and warp ribbon for eventual tactile sensing [160]. Flexible MEMS accelerators embedded in textiles also enable the fabrication of fabric-based wearable devices for recording of body movements [161].

The work of Yang et al. [162] used a water soluble polymer poly(vinyl alcohol), PVA, as sacrificial paste (ecofriendly option) on textile to construct capacitive MEMS cantilever. The PVA polymer was screen-printed from water solutions and dried at 100 °C and the dry film was peeled off at 100 °C, compatible with most textiles. The capacitive cantilever with a resonant frequency of 145 Hz was fabricated entirely using screen printing method. Luo et al. [163] developed a paper-based (cellulose fiber network), low-cost, easy-to-use, wearable tactile sensor array. Their capacitive sensors employed a deformable triangular PDMS sensing membrane having high sensitivity and zero DC power dissipation. Trade-offs among different sensor array designs were investigated to achieve an optimal design. Each sensor in the array demonstrated a nominal capacitance value and sensitivity of approximately 1 pF and 30 fF/mmHg, respectively.

**Figure 16:**
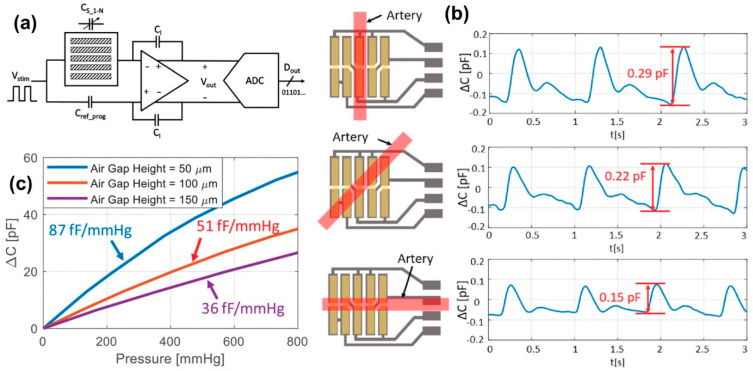
(**a**) Sensor array with CMOS interface circuitry. (**b**) Measured blood pressure pulse waveform under different orientations at the ankle. (**c**) Simulated sensitivity with different airgap sizes. Reprinted/adapted with permission from Ref. [163]. Copyright 2020, IEEE.

An application-specific integrated circuit (ASIC), consisting of a capacitance-to-voltage (C/V) converter followed by an analog-to-digital converter (ADC) was connected to a fabricated sensor array as shown in Figure 16a. When a stimulus voltage pulse, V_stim_, is applied, an output voltage from the C/V converter, V_out_, develops that is proportional to the difference between the sensor capacitance value (C_s_) and a programmable reference capacitor (C_ref_prog_). The C/V converter output voltage, V_out_, is then digitized by the ADC for subsequent signal processing. The effect of a sensor array positioning with respect to an artery is depicted in Figure 16b. The measured blood pressure pulse waveform shows a maximum amplitude when the sensor array is aligned with the artery. The measured blood pressure pulse amplitude decreases to 7.0 mmHg from 29 mmHg, corresponding to a sensor capacitance change of 0.22 pF, when the sensor patch exhibits a 45° angle with respect to the artery. When the sensor patch is perpendicular the pulse amplitude decreases to approximately 5.0 mmHg, which is equivalent to a sensor capacitance change of 0.15 pF as shown in Figure 16b. The authors also used finite element simulation to study the trade-offs between sensitivity and airgaps at the sensor–skin contact. Simulation results indicated that a smaller airgap would result in a higher sensitivity as shown in Figure 16c. Ojuroye et al. [164] tested several hydrophobic encapsulation systems over a tactile sensing e-textile, and tested the performance of the hydrophobized sensor e-textiles against several washing/drying cycles. They reported that the tactile or proximity sensors integrated into the fabric failed after 10 to 15 washing cycles depending on the washing speed and temperature [164]. Such studies should be conducted more often as they may be helpful in developing standards in the future for e-textile components and to develop washability standards for e-textiles [165]. Advances in flexible triboelectric tactile sensors are also moving at high pace [166] and their integration into fabrics or wearables with good durability will be the next challenge. Kirthika et al. [167] fabricated and compared a series of fabric-based tactile sensors using flexible piezoresistive materials and two types of conductive textile materials with variable layer constructions. Their sensors featured high sensitivity, low power consumption and could be operated at different curvy surfaces and dynamic forces [168].

Castano et al. [169] reviewed different processes and components of tactile sensors built into fabrics or textiles. They also studied typical pressure ranges and sensitivity of the devices fabricated. Table 8 summarizes both resistive and capacitive touch sensors integrated into fabrics and almost all of them are compatible with MEMS production technologies as long as proper fabrics are employed [170,171,172,173].

**Figure 17:**
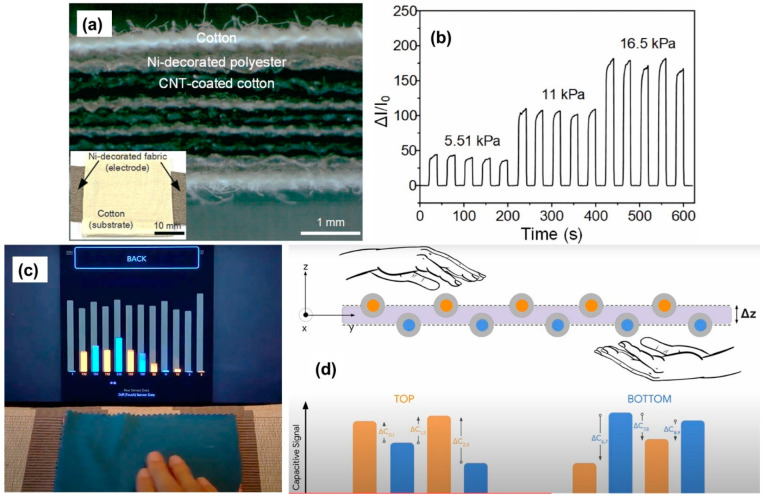
(**a**) Optical microscope image showing cross-sectional view of the sensor. The image clearly shows MCNT-coated cotton/Ni-decorated polyester layers and cotton substrates. Inset is a photograph of as-fabricated sensor. (**b**) Transient responses of the sensor under repetitive loading/unloading cycles for various pressures (5.51, 11 and 16.5 kPa). (**c**) A video snapshot of the operator touching the fabric with four fingers. (**d**) Schematic representation of bottom and top touch action by hand and the relevant capacitive response. The sensor operates stably and exhibits immediate response to the loaded/ unloaded pressure. Reprinted/adapted with permission from Ref. [174]. Copyright 2020, Association for Computing Machinery (ACM).

Figure 17a displays photographs of the fabric sensor with a three-layered structure of MWCNT and Ni modified textile fibers. The alternatively MWCNT-coated cotton/Ni-decorated polyester layers are shown as cross-sectional view of the sensor, and the inset shows the final form of the sensor with the cotton substrate and Ni electrodes. Figure 17b shows the real-time pressure-sensing response of the sensor under repetitive loading/unloading cycles for 5.51, 11 and 16.5 kPa, pressures. The result displays that the sensor exhibits consistent current changes under recurring application of pressures.

A novel dual-sided woven touch sensor that can recognize and differentiate interactions on the top and bottom surfaces of the sensor is shown in Figure 17c [174]. The fabric tactile sensor is based on an industrial multi-layer textile weaving technique, yet it enables a novel capacitive sensing pattern, where each sensing element contributes to touch detection on both surfaces of the sensor simultaneously. Figure 17d shows the process schematically with capacitive intensity depending on the applied pressure by the user’s hand. Unlike the common "sensor sandwich" approach used in other works this tactile sensor inherently minimizes the number of sensing elements, which drastically simplifies both sensor construction and its integration into soft robots, while preserving maximum sensor resolution [175,176]. It has to be pointed out that in complex grasping and manipulative tasks, as those performed in daily activity, such as object stability, slip avoidance and force modulation, are strongly dependent on the tactile feedback distributed on the overall finger and palm surfaces [177]. It must also be mentioned that in performing sensitive grasping tasks with linear sensor response the upper force range does not usually exceed 3–3.5 N, causing losses in the transduction of higher forces. Sensor ranges as high as 7 N with linear response would allow power grip tasks for robots similar to human hands [178].

Haptic tactile perception is an important component for object recognition and handling. However, attributing tactile perception to robotic hands is exceptionally challenging, as it requires active touch assessment involving multidirectional manipulation and sensing. Recent works on wearable mechano-transduced tactile sensors for haptic perception addressed this issue by designing a skin-inspired liquid-based microfluidic MEMS tactile sensor capable of haptic perception. A specific tactile sensor had an elastomeric structure with a microfluidic protrusion [179]. Multidirectional forces applied to the protrusion produced fluid displacements that can be measured electrically. Such an architecture was sensitive to differentiate surface changes below 0.5 mm. Sensors with such MEMS design attributes can be made thin and flexible, with skin adhesion to directly discriminate surface features, contour changes and object stiffness. Such devices are of key importance with potential in assistive robotics.

**Figure 18:**
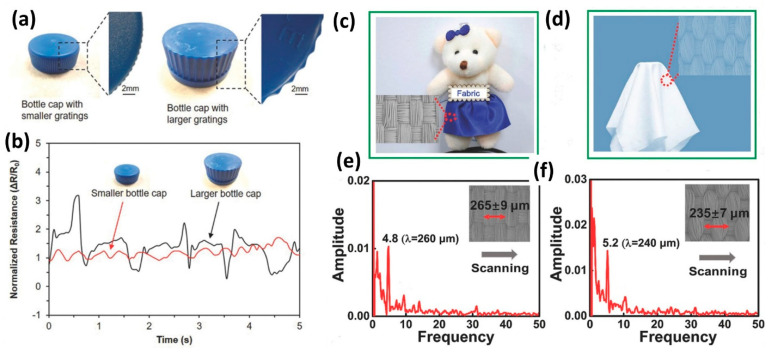
(**a**) Photographs of bottle caps with different size and grating parameters. Inset shows the magnified images of the small bottle cap with 0.5 mm grating height, and the larger bottle cap with 1 mm grating height, respectively. (**b**) Dynamic electrical profile of the sensor scanning across the smaller bottle cap (red), and larger bottle cap (black). (**c**,**d**) The pictures of two fabrics (fabric A and B) that have different warp and weft densities. The inset figures are the SEM images. (**e**,**f**) The FFT wave patterns of fabric A and B, respectively, and the texture dimensions are calculated as 260 and 240 µm by the function f = v/λ. The insets of (**e**,**f**) show the scanning direction and the average dimensions of two fabrics (265 ± 9 and 235 ± 7 µm, respectively) measured from the SEM images. Reprinted/adapted with permission from Ref. [179]. Copyright 2017, Wiley-VCH GmbH.

An example is given in Figure 18. In particular, two different bottle caps, as shown in the figure with the insets detailing the grating features, of ≈ 0.5 mm rounded gratings and ≈ 1 mm triangular gratings, respectively (see Figure 18a). The dynamic resistance profile was registered using a customized LabVIEW program (Figure 18b) in which the cyclic frequency and signal amplitude were consistent to the pitch and grating height, respectively. Unexpected spikes were observed while scanning the larger bottle cap, and this was attributed to the curvature of the bottle cap that should create additional forces as the finger scans across the object [179]. It is remarkable to use such sensors to perceive some more complicated microscale textures, which are very difficult to tell by fingertips. Usually, human fingertips can distinguish the qualitative identification of fabrics with different densities, but cannot quantify the textures. A sensor can be utilized to quantify such information just by scanning over fabrics. A unique study used two different fabrics (Fabric A and B) that had dissimilar warp and weft densities as shown in Figure 18c,d [180]. The sensor could be run over the fabric with a fixed vertical pressure of 200 Pa with a scanning velocity of 1.25 mm s^−1^ so that the characteristic frequency peaks only depend on the surface texture. The frequency domain spectra of fabric A and B are shown in Figure 18e,f, respectively, with characteristic frequency peaks (f) at 4.8 and 5.2 Hz. These can be used to calculate the texture dimensions (v/f = 260.42, 240.38 µm, respectively). The calculated results confirm average dimensions of two fabrics (265 ± 9 and 235 ± 7 µm, respectively) obtained by the SEM images.

For future directions in texture recognition and quantification using flexible wearable MEMS, issues such as the sensor sensitivity limitations due to the presence of particulate contaminants, such as dust and pollen, must be addressed. On real surfaces, these particles are approximately the size and height of textile-embedded sensors (10–100 µm) and thus, can cause significant variation in texture identification regardless of the sensitivity of the sensing element [181]. Tactile sensing helps detect defects on the surface of an object, determine exact gripping force so you can adjust for fragile items, or validate whether one has gripped the correct part, among other applications. MEMS sensing applications are rapidly expanding including pressure fluid regulation and control, optical switching, mass data storage and chemical and biological sensing and control. Adaptation to robotic platforms is also rapidly catching up particularly in the field of micro-sensors that integrate the MEMS sensor with the signal processing circuits on the same chip/platform to produce smart sensors [182].

## 6. MEMS Tactile Sensors on Robotic Platforms: Demonstrators

In closing, we will present and discuss a number of noteworthy studies on MEMS tactile sensors integrated into various robotic platforms of both humanoid and industrial manufacturing robots. In many cases reported so far, two-finger robotic grip platforms have been used, but also MEMS that are integrated into wearable glove-like flexible platforms can be used on humanoid robotic hands [183,184,185]. Table 9 shows a series of robotic platforms that implemented MEMS-based griping and touch sensing sensors.

Soft sensors with strain-insensitive and multimodal features are intriguing due to their high practical relevance. However, incorporating these functionalities into MEMS made of soft materials has been challenging. A number of successful attempts have been demonstrated such as multimodal measurement of proximity and touch force by light- and strain-sensitive multifunctional MEMS sensors [198,199,200]. For instance, an immediate application can be the need for in situ collaborative robots (ISCRs) that are safe enough to operate within a confined space while allowing the collocated workers to retain their sensory presence to use intuitive or an admittance (cooperative) control of the robot [201]. This is considered under the context of force sensing and contact detection of continuum robots.

**Figure 19:**
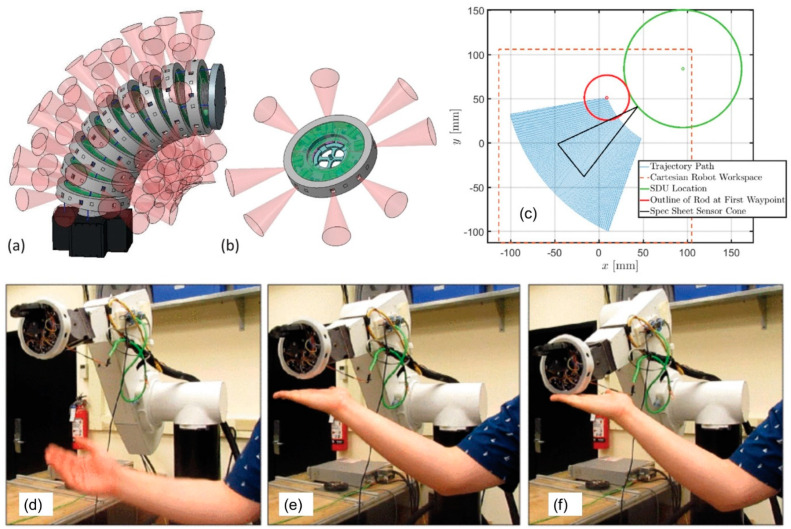
(**a**) Continuum segment augmented with proximity sensing, mapping, force sensing and localized contact detection capabilities. (**b**) Multi-modal sensing disk unit (SDU). (**c**) Trajectory of the Cartesian stage robot during the sensor characterization experiment. Video snapshots showing (**d**) robot moving with constant velocity, (**e**) slowing down near human contact and (**f**) stopping motion after detecting contact. Reprinted/adapted with permission from Ref. [198]. Copyright 2014, IEEE.

Efforts have been made to build MEMS sensors with proximity and force sensing capabilities to augment continuum robots with whole-body mapping and force localization capabilities [198]. Such a multi-modal sensory MEMS attachment is shown in Figure 19. The mechanical architecture of continuum robots is made up of spacer disks that are used as passive elements on which a central backbone is mounted, and through which secondary backbones glide to attain controlled bending in different directions. Augmentation of continuum robots with situational awareness can be performed by substituting passive spacer disks with multi-modal sensing disks units (SDUs), as shown in Figure 19a,b. As such, these MEMS sensors will sense proximity, map their environment, detect and localize contact and sense force. In Figure 19c, the rotary stage was positioned at the corner of the Cartesian robot’s workspace in order to maximize its usable workspace. Afterwards, the SDU was rotated such that its detection cone was entirely within the workspace of the robot. Next, a trajectory was planned to sweep the Delrin rod (a plastic cylinder, red circle in Figure 19c) through the detection cone of the sensor. Starting at a position touching the SDU, but out of the detection cone of the MEMS sensor, the rod was swept at constant radius for ±15° about the center of the SDU. The rod was stopped every 5 mm arc length to collect 20 readings from the sensor. After the full 30°, the sweeping radius was increased by 1 mm and the system started sweeping 30° at constant radius. This process was completed until the center of the rod was 135 mm radially from the SDU outer diameter (the blue zone in Figure 19). The same plot was repeated for different rods featuring different surface features to assess the sensitivity of the experiments [198,199,200]. Eventually, the authors also utilized their robotic arm to detect human touch as shown in Figure 19d–f. Therein, MEMS sensor attached robotic arm utilized the Hall effect touch sensors to stop the robot’s motion when contact was detected, the proximity sensors were used to reduce the robot’s velocity as the bracing surface was being approached to reduce the risk on contact with humans.

A humanoid robot that relies on joint information (i.e., position and/or force) will disregard many of the benefits given by a multimodal sensing device or sensor. For instance, how could a robot differentiate multiple simultaneous touch points? How could it collect more information on object materials or surface structures? As we saw in the previous sections, slippage and surface roughness can be classified by sensing vibrations; temperature changes can be translated into different materials; shear-stress sensors support the recognition of edges; and proximity sensors enable a reaction prior to touching the robot that is especially useful in motion control [202,203,204]. It seems that there is a great need for fitting even the most complicated robotic arms and hands with simply produced but efficient, robust and repeatable MEMs sensors as such devices will not be prohibitively costly but also would take up extra space and be light weight. Paper-based MEMS sensors [205,206,207] are also promising systems that can be integrated into robotic platforms. Ink jet printing has been used to deposit MEMS designs and sensors on paper and thin plastic films that can definitely reduce costs and improve biocompatibility if they can be mounted to robotic platforms effectively [208,209,210,211].

**Figure 20:**
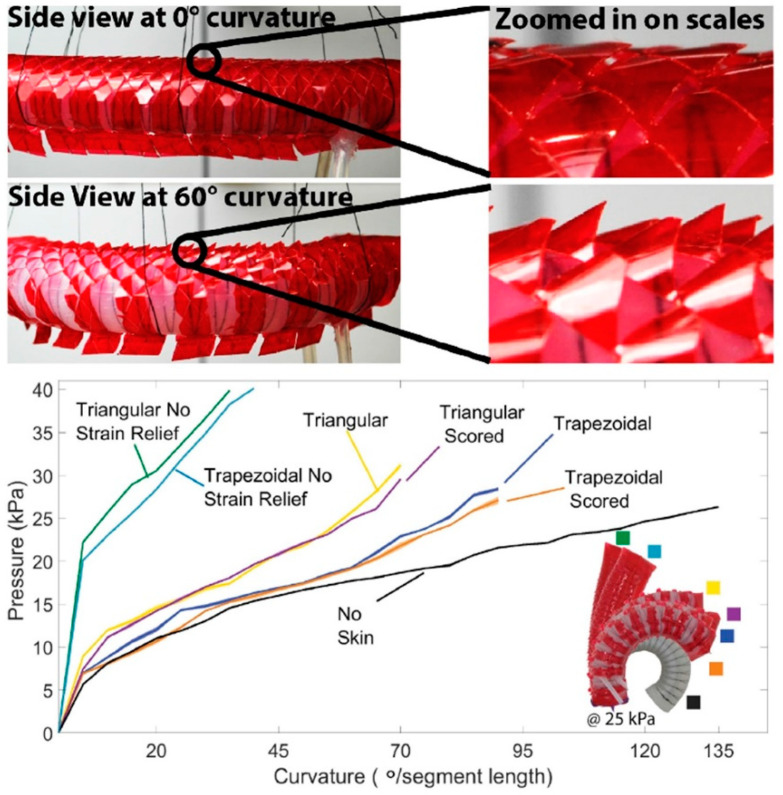
Photographs above: Snake-inspired Kirigami skin for lateral undulation of a soft snake robot with and without curvature. Effect of strain relief designs on curvature vs. pressure relationship. Inset shows the achievable curvature of each skin when the actuator is inflated to the same pressure. Reprinted/adapted with permission from Ref. [219]. Copyright 2020, IEEE.

According to Fujita [212], the MEMS technology is expected to have impacts on robotics in three ways: (1) providing sensors and actuators, (2) introducing a new intelligent system concept, such as an autonomous distributed system, and (3) realizing micro robots. For instance, inspired by the robustness and stability of biological snake locomotion, snake robots carry the potential of meeting the growing need for robotic mobility in unknown and challenging environments. By using the natural motion of snakes, rough and cluttered environments can be traversed with ease [213]. A study of the navigation method for a snake robot based on the kinematics model with MEMS inertial measurement unit (IMU) has been recently reported [214]. Still, there is an excessive struggle in effectively achieving autonomous positioning, especially when the snake robot implements positioning without an external assistant in complex unknown environments. MEMS-IMU systems integrated into snake robots can be effectively used to control and track the snake robot motion tracking system [215]. Snake robots with the ability to measure environmental contact forces acting along their body length have also been reported. Isolated actuators were inserted inside each joint module with custom-designed force/torque sensors so that every section of the flexible body can sense independently [216,217]. Snake robots can be an ideal platform for MEMs-based sensors, as MEMS technologies can be fitted to robot body sections where many degrees of freedom must be manipulated and active sensing of the unknown environmental terrain must be constantly sought [218]. Branyan et al. [219] designed a snake-inspired skin having the advantages of frictional anisotropy without interfering with the deformation required to propagate bends along the soft robot’s body. The principles of Kirigami indicate the activation and deactivation of scales as the soft actuators deform — similar to biological snakes activating their scales to increase friction [219]. Figure 20 shows photographs (top panels) of the artificial snake scales and the relationship between pressure and curvature to demonstrate how the strain relief design allowed for more curvature of the actuator. The four skins tested were compared to an actuator with no skin, and actuators with no strain relief design. Their characterization indicated that the skin did not restrict curvature as expected, but reached moderate curvatures. The actuators without strain relief required larger pressures to reach their maximum curvatures. The maximum curvature achieved by the trapezoidal profile actuators before failure was 90° and for the triangular profile actuators was 70° (Figure 20).

## 7. Summary and Outlook

As we reviewed in this article with many examples, in MEMS, a wide variety of transduction mechanisms can be used to convert real-world signals from one form of energy to another, thereby enabling many different microsensors, microactuators and microsystems. As of today, despite several market stagnation and supply chain problems saddling many electronic components sectors, MEMS still remains a shining star in the semiconductor industry. Opportunities in automotive, consumer electronics, mobile, robotics and medical are on the rise. On one hand, medical applications have been driven mostly by microfluidics, flowmeters, pressure and inertial MEMS. On the other hand, robotics applications were driven by inkjet heads, microbolometers and pressure MEMS [220,221,222,223,224,225]. The market prospect, however, is huge for RF MEMS and oscillators that will be used in next-generation 5G infrastructure. Autonomous robots need dozens of sensors to interact with their surroundings. These sensors also have to be as small as possible so they would not take up space needed for other equipment, interacting with or carrying people and batteries. The automotive industry is consequently one of the fields in which MEMS sensor production and use are expected to grow the most. MEMS are efficiently fabricated at very high volumes using large-scale semiconductor manufacturing technologies. However, these technologies are not feasible for the cost-efficient engineering of specialized MEMS devices at low- and medium-scale dimensions. The 3D printing of MEMS devices and sensors could allow engineered MEMS devices at affordable costs and for custom-built environments [226]. The MEMS technology is expected to have a huge impact on robotics that is manifested in three main streamlines as follows: (1) Effective sensors and actuators, (2) integration of intelligent system concepts, such as autonomous distributed systems, and (3) engineering micro robots [227].

The benefits of autonomous mobility, improved safety, remote operation, remote data collection and improved repeatability are just a few of the reasons why the MEMS devices, the applications of MEMS and NEMS in robotics have been highly popular and were also adapted to flexible and wearable sensor applications as discussed in this work. Review of the robotics literature employing MEMS devices indicates that MEMS sensor platforms particularly developed for pressure and tactile sensing offer a number of indispensable benefits in certain practical applications as follows:

- Very low noise and unique relative accuracy;
- Very low power;
- Improved gyroscope temperature stability;
- Obstacle detection with advanced color and light manipulation;
- Terrain recognition with integrated ultrasonics;
- On board instant IMU data computation;
- Easier and faster robot motor control;
- Noise filter and noise cancellation;
- Robot operating system drivers for all on-board sensors.

Very recent advances in MEMS-based barometric sensors have already paved the way for their integration into advanced robotics applications. For instance, such devices combine a barometric pressure and a temperature sensor in small enclosures such as 2.0 mm × 2.0 mm × 0.8 mm packages with multiple input voltage levels including 1.2 V, 1.8 V and 3.3 V. At the same time, new studies have been published to improve fundamental micro fabrication production technologies for MEMS on fabrics/textiles using robotic applications. This will result in a cheap, easy-to-design, flexible, rapid means to manufacture multifunction smart textiles/garments for a large set of robotic platforms. Fabrication processes include thick film printing and sacrificial etching as well as inkjet printing.

Low-cost, solid-state MEMS accelerometers will be indispensable short duration distance-measuring devices for a mobile platform or robot. These MEMS can be combined with gyroscope and odometer to form a dead reckoning positioning system for a mobile robot or platform. In a real world application of the MEMS accelerometer, the gravitational component needs to be compensated due to a change of orientations of sensor sensitive axes [228]. For low cost applications, several MEMS accelerometers can be used to resolve for both the gravitational and translational accelerations. Further research is being conducted on the suitable modeling of the accelerometer in order to reduce the effect of random bias drift. We also reviewed the performance of MEMS-based stiffness sensors having added advantages of closed-loop operation and the stiffness-adjusting mechanisms, making these devices better candidates for use in high-precision and high-bandwidth force measurement applications. In recent years, a new sensing approach, which utilizes 2 degree-of-freedom (DoF) weakly coupled resonators, has been proposed. By measuring the mode shape changes instead of the frequency shifts, it has been shown that this type of sensing devices has: 1) orders of magnitude higher sensitivity than conventional single DoF resonator sensors; 2) common mode rejection capabilities. New MEMS sensors for stiffness change sensing applications based on three weakly coupled resonators have also been reported with 50 times better sensitivity to stiffness changes compared to 2-DoF counterparts. On the other hand, in view of the extensive increase in flexible devices and wearable sensor technologies, the development of polymer MEMS is becoming more and more central even though, polymer micromachining for MEMS sensors is not yet as mature as the Si MEMS. Innovative ink printing, polymer etching, layer by layer deposition methods are enabling new polymer MEMS sensors such as bilayer polymer double-clamped resonators with integrated piezoresistive readout capabilities [229].

Finally, we discussed triboelectric driven MEMS sensors with nanogenerators (NGs) relying on the piezoelectric and triboelectric effects to convert mechanical energy in our living environment into electricity for powering robotic MEMS sensors [230]. New studies will focus on coupling triboelectric generators with MEMS for safety in robots such that a triboelectric generator yields voltage when it receives a mechanical impact. The voltage is proportional to the mechanical impact and when the voltage exceeds a certain level, a MEMS sensor can engage and can disconnect the current in a safety electronic system [231]. MEMS sensors will play a major role in robotics technology. Because information itself has no mass and size, the smaller smart machines are better suited to gathering, handling and transporting and information storage. MEMS are indispensable to the future infrastructure for sensor data exchange and storage because they have information processing capability in themselves. MEMS can also contribute to improving the quality and density of display and sensing [232,233,234].

This review presented the main aspects that are essential to mimicking human’s sense-of-touch mechanism when building multifunctional electronic skin and haptic systems with MEMS technologies. This can be achieved by studying the fundamental theories about human skin and the role of mechanoreceptors in tactile sensation. MEMS integrated into flexible platforms carrying typical design structures of mechanoreceptors with the basic requirements needed for tactile sensing are still challenging. This review also highlighted recent studies on some techniques that are employed for tactile transduction, the state-of-the-art and novel materials commonly used for sensing and potential applications of MEMS tactile sensors, specifically towards building the human’s sense-of-touch for soft, micro- and snake robots.

## Figures and Tables

**Table 1:** Classical modes (excluding MEMS) of tactile sensors. Reprinted/adapted with permission from Ref. [25]. Copyright 2013, Elsevier.

Modality	Sensor Type	Advantages	Disadvantages
Normal pressure	Piezoresistive array	Simple signal conditioning, mass production adaptable	Temperature sensitive, reproducibility issues and signal drift
	Capacitive array	Good sensitivity	Complex circuitry required
	Optical	No interconnects to break	Requires on board computation devices for applied force
Skin deformation	Optical	Compliant membrane and no electrical interconnects to be damaged	Complex computations required and needs customized hand design
	Magnetic	Array to hall-effect sensors possible	Complex computations required and needs customized hand design
	Resistive Tomography	Good coverage, manufacturability and robustness	Poor spatial resolution
	Piezoresistive (curvature)	Directly measure curvature	Frailty of electrical interconnects
Dynamic tactile sensing	Piezoelectric (stress rate)	High bandwidth	Frailty of electrical junctions
	Skin (vector) acceleration	Simple	Gets complicated for large spatial mapping

**Table 2:** Evolution of MEMS from the early 1950s.

Year	1st Milestone	2nd Milestone	3rd Milestone	4th Milestone	5th Milestone
1950s	1958 Silicon strain gauges commercialized	1959 Richard Feynman issues a challenge to make an electrical motor smaller than 1/64th of an inch.	n/a	n/a	n/a
1960s	1961 First silicon pressure sensor fabricated	1967 Invention of surface micromachining and Resonant Gate Field Effect Transistor, (RGT).	1968 Development of sacrificial materials to free MEMS from the silicon substrate.	n/a	n/a
1970s	1970 First silicon accelerometer demonstrated	1979 First micro machined inkjet nozzle	n/a	n/a	n/a
1980s	1980–1982 First experiments in surface micro machined silicon.	1982 Disposable blood pressure transducer	1982 Silicon etching standards established	1982 LIGA process (fabrication of high-aspect-ratio microstructures)	1988 First MEMS conference
1990s	1992 First micro machined hinge	1993 First surface micro machined accelerometer sold (Analog Devices, ADXL50)	1994 Deep Reactive Ion Etching is patented	1995 BioMEMS emerge and grow fast	
2000s	2000 MEMS optical-networking components industrialized in large scales	2010 MEMS adapted to handheld electronic devices	2015 MEMS adapted to wearable and human centric technologies	2020 MEMS as smart sensors and MEMS in robotic servants	

**Table 3:** Properties of selected flexible pressure sensors with different sensing mechanisms *.

Sensor Type	Active Layer	Sensitivity (kPa^−1^)	Detection Limit (kPa)	Reference
Capacitive	Ti_0.91_O_2_ NSs/BC paper	2.44 × 10^−3^	166	[72]
Capacitive	MEMs-based film	7.10 × 10^−4^	1	[73]
Capacitive	Bump PDMS film	6 × 10^−3^	250	[74]
Capacitive	Thin PDMS film	6.13 × 10^−3^	45	[75]
Capacitive	Bump PDMS film	0.03	430	[76]
Capacitive	Electrolyte/filter paper	0.04	19	[77]
Resistive	MWCNTs/cotton cellulose	0.02	20	[78]
Resistive	Graphene/polyimide	0.18	2	[79]
Resistive	LSG/GO film	0.96	50	[80]
Piezoresistive	SCNTs/cellulose nanofibrils	4.40	0.5	[81]
Piezoresistive	Graphene/cellulose paper	9.83	1.7	[82]

* Note. PDMS = Polydimethylsiloxane, MWCNTs = Multi-walled carbon nanotubes, LSG = Laser-scribed graphene, GO = Graphene oxide, SCNTs = Sulfonated carbon nanotubes.

**Table 4:** Common applications of flexible pressure sensing and ranges encountered.

Application	Pressure
Vascular pulse (75–150 mmHg)	10–20 kPa
Human fingertip texture, shape sensing	10–40 kPa
Hand grip	0–100 kPa
Fingerprint sensor	1–2 kPa
In-shoe pressures	<1 MPa
Tactile robotics	10–100 kPa

**Table 5:** Comparison between the capacitive pressure sensors based on PDMS microstructure. DIW indicates deionized water.

Material/Structure	Sensitivity (kPa^−1^)	Range (kPa)	Response/Recovery Time	Minimum Detection	Reference
PDMS/Wrinkled microstructure	0.0012 4.2 × 10^−6^	<1 >8	578/782 ms	n/a	[84]
Porous PDMS	0.26 0.01	0–0.33 0.33–250	15/− ms	1 Pa	[85]
Porous PDMS	0.046 0.051	0.01–0.05 0.1–0.5	NA	5 Pa	[86]
Bubble trapped PDMS	5.5 × 10^−3^	0–10.20	~351/386 ms	NA	[87]
PDMS/DIW	0.068 0.095	0.01–0.05 0.1–0.5	~110/110 ms	1 Pa	[88]

**Table 6:** Efficient resonant action pressure sensors performance comparison.

Basic Accuracy	Output Conformity Error	Hysteresis Error	Repeatability Error	Sensitivity (Hz/kPa)	Range (kPa)	Operating Temperature (°C)	Reference
n/a	0.021	n/a	n/a	7.35	<250	−40 to +60	[103]
n/a	0.021	n/a	n/a	20	<180	−20 to +80	[108]
n/a	n/a	n/a	n/a	45	<110	n/a	[109]
0.045	0.0102	0.0045	0.044	n/a	<400	−20 to +60	[107]
0.064	0.0148	0.0525	0.0315	36.58	<100	−20 to +60	[107]

**Table 7:** The piezoelectric properties of PVDF, P(VDF-TrFE) nanofibers and their nanocomposite via electrospinning. The table is compiled from data in [131].

Nanofiber Substrate	Operation Voltage/Resistance	Applied Current	Sensitivity	Detection Limit	Cyclic Stability	Reference
PVDF	140 mV	n/a	42.00 mV/N	n/a	n/a	[132]
PVDF	1–2.6 V	1.4–4.5 μA	n/a	n/a	n/a	[133]
PVDF/PET and PDMS	100 mV at 0.025 MPa	n/a	5.812 mV kPa^−1^	n/a	n/a	[134]
PVDF	~3 mV	n/a	n/a	n/a	n/a	[135]
PVDF/MWCNT	6 V	n/a	the volume conductivity is 5 orders higher than pure PVDF nanofibers	n/a	n/a	[136]
PVDF-0.05MWCNT-0.1OMMT	58 ± 2.5 mV 48 ± 4.7 mV (pure PVDF)	n/a	10.9 ± 1.25 mV/N 8.84 ± 1.57 mV/N (pure PVDF)	n/a	n/a	[137]
AgNWs doped PVDF	n/a	n/a	29.8 pC/N (for d_33_) 18.1 pC/N (pure PVDF)	n/a	n/a	[138]
PVDF/PPy	1.6 S·cm^−1^ 3.2 × 10^−16^ S·cm^−1^ (pure PVDF)	n/a	40-fold increase in the relative conductivity	n/a	n/a	[139]
PVDF/PPy	10^7^ Ω·cm 10^17^ Ω·cm (pure PVDF)	n/a	200 Ω·cm/Pa 20 Ω·cm/Pa (pure PVDF)	<0.02 MPa	>25	[140]
P(VDF-TrFE)/PI	0.5–1.5 V	6–40 nA		<0.1 Pa	1000	[141]
P(VDF-TrFE)/PDMS	~2000 mV	n/a	120 mV/µm		>1000	[142]
P(VDF-TrFE)	~5 mV	n/a	60.5 mV/N	n/a	n/a	[143]
P(VDF-TrFE)	~0.7 V	n/a	n/a	n/a	n/a	[144]
P(VDF-TrFE)	n/a	n/a	15.6 kPa^−1^	1.2 Pa	100,000	[145]
P(VDF-TrFE)/PDMS-MWCNT membrane	25 V (triboelectric voltage) 2.5 V (piezoelectric voltage)	~6.5 μA (triboelectric current) ~2.3 μA (piezoelectric current)		n/a	n/a	[146]
P(VDF-TrFE)	300 ± 5 mV	n/a	n/a	n/a	n/a	[147]
P(VDF-TrFE)	n/a	n/a	110.37 pC/Pa	n/a	n/a	[148]
P(VDF-TrFE) (3D sensor)/PDMS	>1200 mV (flat shape) ~1000 mV (wrist shape) ~500 mV (finger shape)	n/a	23 VN^−1^ (flat shape) 20 VN^−1^ (wrist shape) 12 VN^−1^ (finger shape)	n/a	n/a	[149]
P(VDF-TrFE) (shell)-PVP/PEDOT: PSS (core)	>1.6 V	n/a	4 mV/mmHg	n/a	n/a	[150]

**Table 8:** A summary of fabric integrated tactile sensors, materials and typical performance data discussed and reviewed by Castano et al. [169].

Fabrication	Components	Sensitivity	Pressure Range	Size	Comments
Embroidery	Conductive thread	Switching threshold	Contact sensing	mm^2^–cm^2^ range	Electrical contact
Patterned electrodes	Conductive ink	0.214 V/pF	0–13 kPa	32 mm^2^	Thickness compression
Surface touch	PEDOT Nylon	0.02 pf/mm	0–2 Pa	Diameter = 5 cm	Capacitance fingers/surface
Laminated electrodes	Thin film deposited metals	0.01 ΔC/mN	0–50 N/cm^2^	Diameter = 250 µm	Capacitance at intersecting points
3D textile capacitor	Conductive fabric 3D textile	2 pF/N/cm^2^	0–0.75 N/cm^2^	9 cm^2^	Thickness compression
Crosslite^TM^ capacitor	Silver-coated textile PCCR	0.05 pF/N/cm^2^	0–30 N/cm^2^	100 mm^2^	Thickness compression
Switch tactile sensor	Plated fabric Cu, Ni	Threshold at 500 g/mm^2^	70–500 g/mm^2^	8 mm^2^	Active sensing cells
Tooth structured	Conductive fabric	2.98 × 10^−3^ kPa^−1^	–2000 kPa	760 mm^3^	Strain in under pressure fabric
Polyurethane foam	PPy-Polyurethane	0:0007 mS/N	1–7 kN/m^2^	4 cm^3^	Conductance increases with compression
Conductive Rubber-based	Carbon polymer	250 Ω/MPa	0–0.2 MPa	9 mm^2^	Resistance changes with applied load
QTC-Ni-based	Pressure composite	~106 Ω/1% compression	25% compression	Diameter = 5.5 mm	Switching behavior

**Table 9:** Selected examples of MEMS-based sensors for object griping and recognition on various robotic platforms.

MEMS Elements	Operation Principle	Robotic Platform	Touch/Grip Object Type	Sensitivity	Reference
Optical fiber	Bragg’s grating	Four finger gripper	Metal, rubber, plastic	139 nm/N	[186]
Beam deformation strain gauge	Wheatstone bridge	Manufacturing robotic arm	None/Torque sensor	1–3 mV/Nm	[187]
Optical/magnetic	Retroreflective markers/Electromagnetic field	Two finger gripper	3D printed plastics	0.01/0.6 mm/deg.	[188]
Resistive sensors	Conductivity changes	Master-slave robotic hand system	Plastics and metals	0.1 N	[189]
Graphene/Nanosilver electrodes	Nanoparticle/elastomer composite resistance change	Humanoid robotic hand	Ceramics, plastics	1.32–3.40% kPa^−1^	[123]
Capacitive/pneumatic	pneumatic deformation sensing	Two finger gripper	3D printed soft plastics	0.03 N	[190]
Resistive/magnetic	Capacitance/magnet displacement	Two finger gripper	Metal, wood plastic	n/a	[191]
Resistive	Nanoparticle/elastomer composite resistance change	YuMi robot	Skin-like soft rubbers	18.83% N^−1^	[192]
Magnetic/barometric	Liquid metal sensing/electrical resistance	Two finger gripper	Plastic objects	85% accuracy	[193]
Resistive	Conductive foam compression	Two finger gripper	Metals, rubber, wood	1.196%/°C and 13.29%/kPa	[194]
Resistive	Resistance change under pressure	anthropomorphic artificial hand	Rigid objects	0.47, 0.45, 0.16 mV/mN for the *x*-, *y*- and *z*-directions	[195]
Tribolectric nanogenerator	Electrostatic induction	Three finger gripper	Plastic, fruits, aluminum, paper	98.1% accuracy	[196]
Resistive	Resistance variation upon compression	Soft robotic hand	100 objects of all sorts	94% basic grasping, 50–80% identification-grasping	[197]

## Data Availability

Not applicable.
